# Interrupted Intramolecular Hydroaminomethylation of *N*-Protected-2-vinyl Anilines: Novel Access to 3-Substitued Indoles or Indoline-2-ols

**DOI:** 10.3390/molecules27031074

**Published:** 2022-02-05

**Authors:** Frank Hochberger-Roa, Perla H. García-Ríos, José G. López-Cortés, M. Carmen Ortega-Alfaro, Jean-Claude Daran, Maryse Gouygou, Martine Urrutigoïty

**Affiliations:** 1 Laboratoire de Chimie de Coordination (LCC), Centre National de la Recherche Scientifique, Université de Toulouse, 31030 Toulouse, France; frank.hochberger@gmail.com (F.H.-R.); Per.Haidee_312@hotmail.com (P.H.G.-R.); jean-claude-daran@lcc-toulouse.fr (J.-C.D.); gouygou@lcc-toulouse.fr (M.G.); 2 Instituto de Química, Universidad Nacional Autónoma de México, Circuito Exterior, Ciudad Universitaria, Coyoacán C.P., Ciudad de Mexico 04510, Mexico; jglcvdw@unam.mx; 3 Instituto de Ciencias Nucleares, Universidad Nacional Autónoma de México, Circuito Exterior, Ciudad Universitaria, Coyoacán C.P., Ciudad de Mexico 04510, Mexico; carmen.ortega@nucleares.unam.mx

**Keywords:** interrupted hydroaminomethylation, indole, indoline-2-ol, *N*-protecting-2-vinyl aniline carbonylation, rhodium

## Abstract

A new synthetic alternative to the synthesis of 3-methyl indoles and 3-methyl indoline-2-ols with an excellent atomic economy is presented in this study. It is demonstrated that the intramolecular interrupted hydroaminomethylation (HAM) reaction is a powerful tool for the formation of these compounds, which exhibit wide-ranging biological activity. Several *N*-Protected-2-vinyl anilines were synthesized and involved in the reaction producing the corresponding 3-methylindole or 3-methyl indoline-2-ol depending on the nature of the *N*-protecting groups.

## 1. Introduction

**Figure 1:**
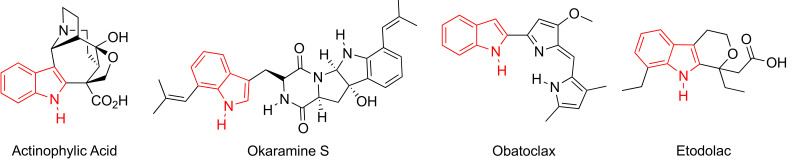
Privileged natural products and pharmaceutics containing indole rings.

The indole ring system is one important motif widely present in nature [1,2,3]. With great structural diversity, many drugs and bioactive compounds include this ring (Figure 1) [4,5,6]. Thus, it is not surprising that synthetic pathways to these heterocycles have been developed and reported in several reviews [7,8,9]. Nevertheless, the importance of indoles as structural elements in natural products and pharmaceuticals is reflected in the perpetual development of new synthetic methods. 

**Scheme 1:**
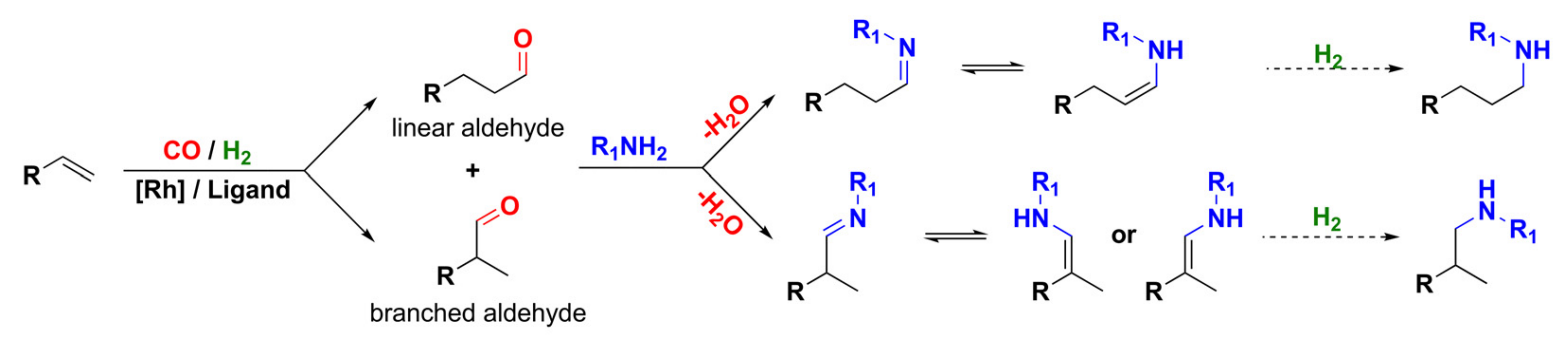
Interrupted HAM (with no hydrogenation step).

Since most of the conventional methods to yield this heterocycle require highly functionalized starting materials and suffer from low atom economy and harsh reaction conditions, transition metal catalysis has become an excellent alternative for the production of the indole. Some syntheses catalysed by Pd [10,11,12], Ru [13,14], Rh [15,16,17], Au [18], Co [19], or Zn [20] have been normally described as more efficient than classical methods with regard to the yield and atom economy. Nevertheless, the reports stand limited, and this field remains little explored. Among the alternatives using transition metals to produce nitrogen containing molecules in one pot, hydroaminomethylation reaction (HAM) [21,22,23] appears to be an excellent strategy. Indeed, the reaction catalysed by rhodium has proved to be a very effective synthetic route for the formation of amines through olefins. This one-pot, atom-efficient reaction consists of hydroformylation of olefin to an aldehyde and then the subsequent formation of an enamine or imine, followed by hydrogenation. Theoretically, the interrupted version should be a method for the access to imines and enamines (Scheme 1). 

In connection with our work on the HAM reaction [24], we envisaged that indole moiety could be obtained via an intramolecular pathway by interrupting the HAM reaction before the hydrogenation step of the enamine formed. Therefore, we studied the interrupted HAM from 2-vinyl aniline substrates in order to obtain the corresponding indoles.

## 2. Results and Discussion

**Scheme 2:**
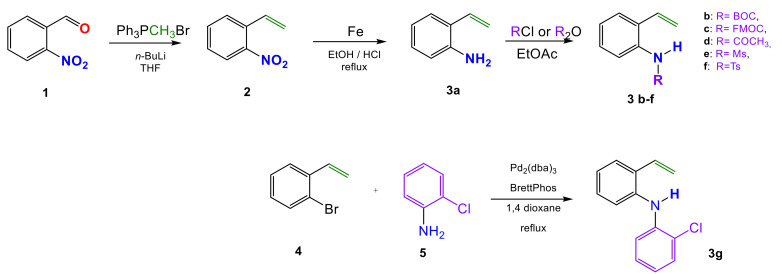
Synthetic pathway for a family of 2-vinylanilines **3a** and **3b**–**g**.

Starting from 2-nitrobenzaldehyde (**1**), 2-vinyl aniline (**3a**) was synthetized in two steps according to a procedure adapted from the literature [25]. Then, *N*-protected vinylanilines (**3b**–**f**) were obtained with good yields (82–92%), as described in Scheme 2.

On the other hand, *N*-(2-chlorophenyl)-2-vinyl-aniline (**3g**) was prepared via Pd-catalyzed condensation of 2-bromostyrene (**4**) and 2-chloroaniline (**5**), as already described in the literature [26].

**Scheme 3:**
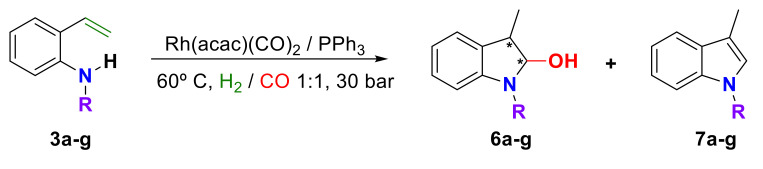
Interrupted intramolecular HAM reaction.

Products **3a** and **3b**–**g** were subsequently involved in the interrupted intramolecular reaction HAM under the same conditions previously developed by our group during a study of the hydroformylation reaction [27] (Scheme 3). All results obtained are recorded in Table 1.

After a total conversion for all 2-vinylanilines, except for *N*-BOC-2-vinylaniline (**3d**) for which the conversion reached is only 40%, chemoselectivity and regioselectivity were good to excellent for the other 2-vinylanilines. Chemoselectivity depends on the nitrogen protecting group. Indeed, only 3-methyl indoles (**7a** and **7g**) were obtained quantitatively from the non-protected *N*-2-vinylaniline (**3a**) and *N*-2-chlorophenyl 2-vinylaniline (**3g**) (Entries 1, 2). Surprisingly, in cases of *N*-mesyl and *N*-tosyl-2-vinylanilines (**3e** and **3f**), only the corresponding indoline-2-ol compounds (**6e-f**) were reached (Entries 6, 7). This behavior could be attributed to the best electron withdrawing character of mesyl and tosyl groups that avoid the dehydration process. This inductive effect can be correlated with pKa [28] for the corresponding organic acid of protecting groups. With this in mind, we employed the chemicalize tool developed by ChemAxom Company [29,30] to predict the pKa of these compounds. The results obtained are included in Table 1.

On the other hand, in the presence of the acetyl, Fmoc and BOC protecting groups with lower pKa, a mixture of products (**6**) and (**7**) was formed with (**6**) in greater proportion of 77 to 85% yield (Entries 3, 4, 5). Nevertheless, the (**6b**–**d**) derivatives readily release the protecting group leading the corresponding skatole.

The indoline-2-ol compound (**6**), which is a five-membered cyclic hemiacetal, is revealed to be stable and appears to be the intermediate product for the formation of indole after dehydration in the interrupted HAM reaction. Indeed, when the reaction of *N*-mesyl- and *N*-tosyl-2-vinylaniline (**3e** and **3f**) was performed in the presence of 1 eq. of p-toluenesulfonic acid, the corresponding 3-methyl indoles (**7e** and **7f**) were quantitatively obtained. This behavior reveals that, in the presence of a lower pka of the corresponding organic acid, we can expect the best electron withdrawing effect of the protecting group on aniline, which contributes to the stability of the indoline-2-ol obtained and avoids the dehydration step.

**Scheme 4:**
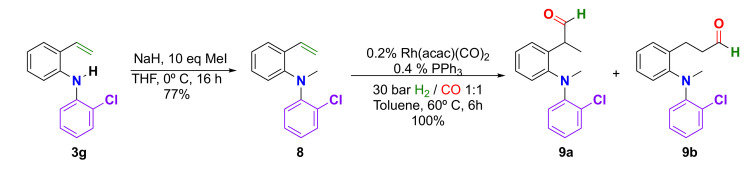
Synthesis and hydroformylation reaction of *N*-methyl-*N*-2-chlorophenyl 2-vinylaniline (**8**).

The formation of compound (**6**) arises from the branched aldehyde formed regioselectively in the hydroformylation reaction, which is the first step of the HAM reaction. To demonstrate this regioselectivity, we studied the hydroformylation reaction of *N*-methyl-*N*-2-chlorophenyl 2-vinylaniline (**8**), a tertiary amine to prevent the condensation of the amine with the aldehyde formed. The reaction was carried out in the same conditions used before (Scheme 4).

The conversion was complete and the two branched and linear aldehydes (**9a**) and (**9b**), respectively, were reached with the branched one as the major product in the ratio of 85:15 (**9a**/**9b**). To the best of our knowledge, the branched aldehyde (**9a**) is a new compound. It was characterized by NMR, and its structure was confirmed by X-ray diffraction analysis (Cf Supplementary Materials S2).

**Scheme 5:**
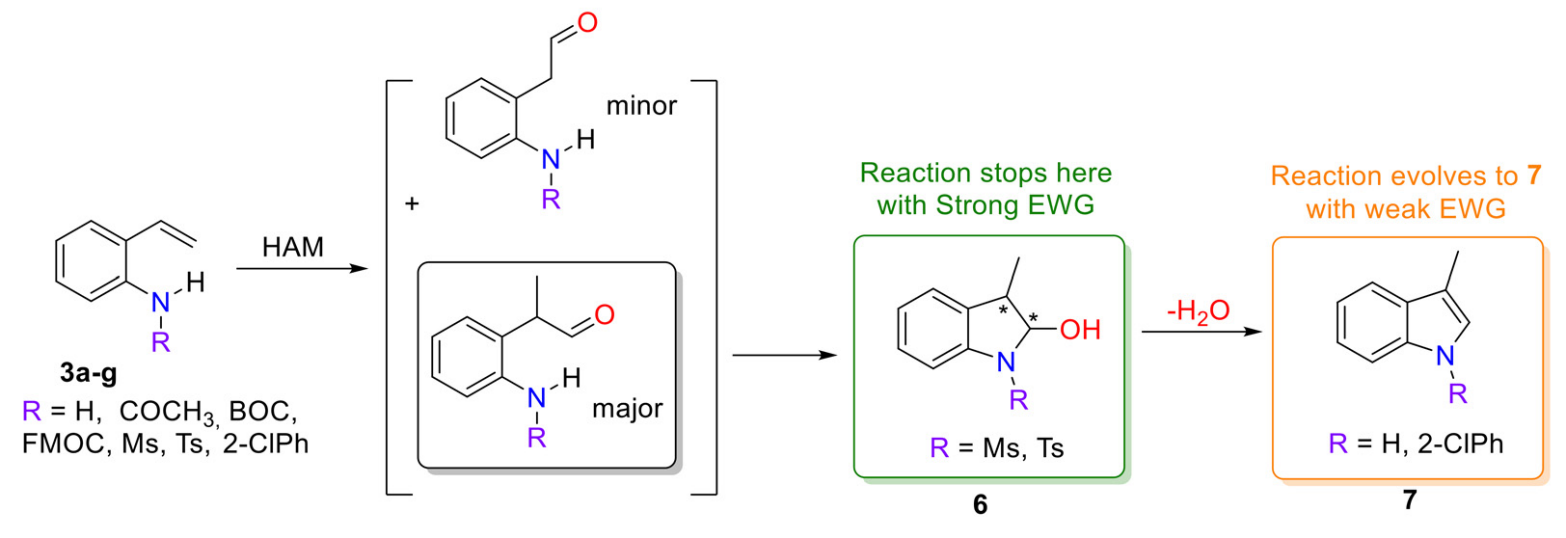
Proposal pathway to the formation of (**6**) and (**7**).

This experiment showed that once the branched aldehyde is formed, the condensation with the secondary amine occurs quickly to afford the indoline-2-ol intermediate (**6**), which can be converted to indole (**7**) after dehydration (Scheme 5). The dehydration process is promoted by weak electron withdrawing groups (phenyl ring or BOC, Fmoc) on the nitrogen atom, conducting the aromatic indole ring. Reactivity can be modified by using strong electron withdrawing groups such as tosyl (Ts) or mesyl (Ms) groups, for which the electron pair of N-atom is less available acting as a conventional amide or sulfonamide and, thus, stopping the aromatization step [31]. 

**Figure 2:**
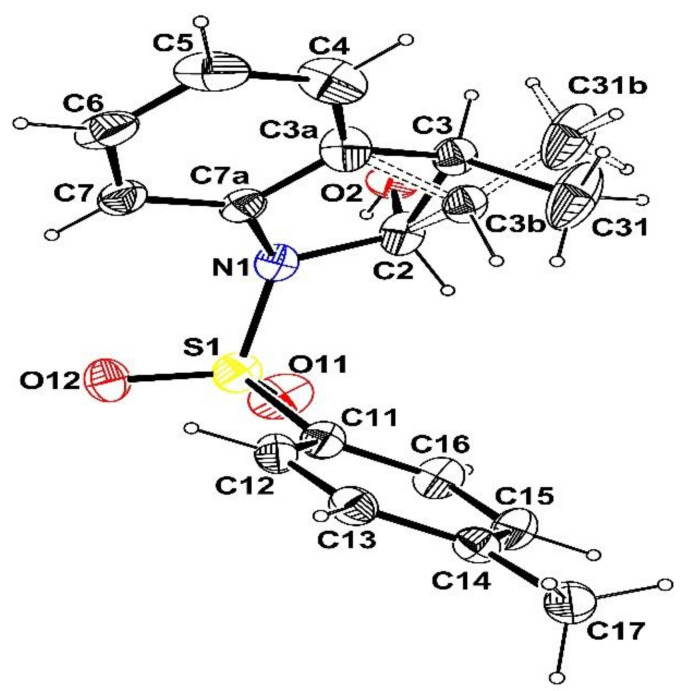
Molecular view of compound **6f** with the atom labelling scheme. Ellipsoids are drawn at the 30% probability level. H atoms are represented as small circle of arbitrary radii. The minor component of the disorder is represented with dashed bonds.

During the formation of the indoline-2-ols (**6**), two stereogenic carbons are created with a diastereoselectivity ratio of 4.5:1 or 5:1 (Table 1, Entries 3, 4, 6, 7). The two cis and trans diastereomers of compound **6f** could be isolated after purification by column chromatography, and they have been fully characterized by NMR and mass spectrometry, but all attempts to crystallise these isomers failed. Surprisingly single crystals suitable for X-ray diffraction analysis were grown from the diastereoisomeric mixture. The two diastereomers co-crystallised in a 70:30 ratio in the unit cell, the major diastereoisomer being the trans isomer (Cf Supplementary Materials S3). Indeed, the molecular structure of **6f** presented in Figure 2 shows that in the (N1 C2 C3 C3a C7a) ring, the C3 atom bearing the methyl group is disordered over two positions in the ratio of 70:30, resulting in the inversion of configuration on C3 (C3b). 

**Scheme 6:**
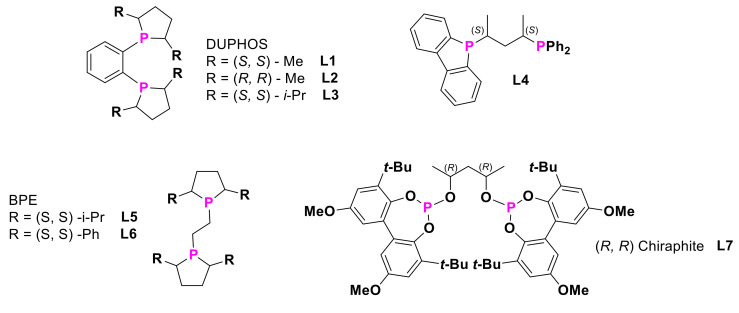
Chiral diphosphines used in interrupted intramolecular HAM reaction of **3f**.

Chiral 2-substituted and 2,3-disubstituted indolines derivatives represent an interesting backbone as they are found in many natural products and biologically active products [32,33,34,35]. Thus, in order to obtain the indoline-2-ol **6f** with good enantioselectivity and diastereoselectivity, we carried out some experiments with *N*-tosyl-2-vinylaniline **3f** and several chiral diphosphine ligands known to be efficient in hydroformylation reactions (Scheme 6) [36].

**Scheme 7:**
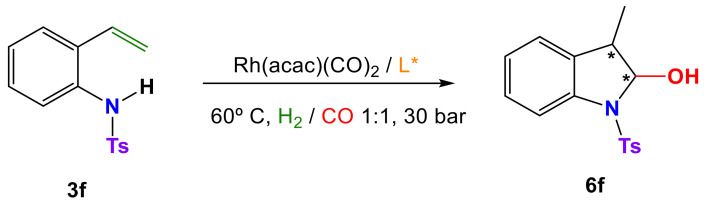
Interrupted intramolecular HAM reaction of **3f** with chiral diphosphines.

The reaction was performed under the same conditions used before (Scheme 7), and the results are reported in Table 2. In almost all cases, the conversion was excellent with values between 95 and 99%. The diastereomeric ratio obtained turned out to be constant with values between 2:1 and 3:1 for **L1-L5** and **L8**, values lower than that obtained with the PPh_3_ ligand (Entries 2–6, 8 vs. Entry 1). Fortunately, with the (S,S)-diphenyl-BPE ligand (**L6**, entry 7), an excellent d.r. ratio of 10:1 was attained. The analysis of the enantiomeric excess (ee) of the major trans diastereomer was performed by chiral HPLC. In addition, the best performance was afforded with the **L6** ligand as chirality inductor improving the ee up to 43%. By contrast, no enantioselective control occurred for the cis diastereoisomer as a racemic mixture was obtained in all cases. Unfortunately, the d.r. of the reaction and the ee of the trans diastereomer could not be improved by driving the reaction at lower temperatures.

## 3. Conclusions

In summary, intramolecular interrupted HAM provides an efficient alternative approach for the synthesis of 3-methyl indoles and 3-methyl indoline-2 ol derivatives in one step. *N*-protecting- 2-vinylaniline derivatives were converted to the corresponding indoles or indoline-2-ols in good to excellent yields. It was found that the chemoselectivity of the reaction was driven by the nature of the protecting group on the nitrogen atom. The presence of tosyl and mesyl groups, which have lower pKa, generates selectively the formation of the corresponding 3-methyl-indoline-2-ols in diastereomeric ratios up to 5:1. In the presence of weak electron, withdrawing groups such as phenyl group, or no protecting group, causes the 3-methyl indole to be exclusively formed. The asymmetric interrupted HAM reaction of the *N*- tosyl(toluenesulfonyl)-2-vinyl-aniline performed in the presence of (S,S)-diphenyl-BPE ligand proved to be highly diastereoselective (d.r. 10:1) but moderately enantioselective (43% ee) for the trans *N*-tosyl-3-methyl-indoline-2-ol isomer. 

Further investigations on the reaction are in progress to design more versatile and efficient catalysts for the stereoselectivity of the reaction to obtain chiral 2,3-disubstitued indoline subunit, which is found in a wide range of natural products. 

## 4. Materials and Methods

### 4.1. General Remarks

All reagents were used as received unless otherwise noted. All reactions requiring a dry and inert atmosphere were performed in Schlenk tubes under Argon.

THF, toluene, and pentane were dried under N_2_ using a solvent purification system (SPS).

^1^H and ^13^C NMR spectra were obtained in CDCl_3_ or CD_2_Cl_2_ at 25 °C on a Bruker AVANCE spectrometer (Bruker Corporation (Karlsruhe, Germany) at the following frequencies: 400 MHz (1H) and 100 MHz (13C). All ^1^H NMR experiments are reported in ppm downfield of TMS and were measured relative to the signals for chloroform (7.27 ppm). All ^13^C NMR spectra were reported in ppm relative to residual chloroform (77 ppm). Mass spectra analyses were performed on API-365 spectrometer (ESI) and on a TSQ 7000 Thermoquest instrument (DCI). High resolution mass spectra (HRMS) were recorded using a Waters Xevo G2 QTof instrument ((DCI) (GCT Premier, Waters-Micromass, Manchester, UK). Gas chromatographic analyses were performed on Clarus 580 Perkin Elmer gas chromatography instrument (Perkin Elmer, 91661 Villebon sur Yvette, France) equipped with a split/splitless injection port (200 °C) and flame ionization detector (250 °C) fitted with an FID detector using an Elite RTx-5 Amine capillary column (Perkin Elmer, Villebonsur Yvette, France) (30 m length, 0.53 mm inner diameter and 0.1 µm film thickness) at 200 °C during all experiments. Chiral HPLC UPC^2^ analyses were performed on Bruker apparatus using Chiralpak AS-3 (Waters, 78280 Guyancourt, France) (4.6 × 100)mm 3 µm N°20523 (Daicel Corp) column, isocratic gradient 98 CO2/2(CH_3_CN + 0.1% HCOOH), flow rate 2 mL/mn, and λ = 250 nm.

The NMR spectra of the main compounds are given in the Supplementary Materials. To predict the pKa of the corresponding organic acids, we used the Chemicalize tool developed by ChemAxon [29,30]

### 4.2. Synthesis

#### 4.2.1. Synthesis of 2-nitrostyrene (**2**)

Adapted from reference [37]. To a solution of Ph_3_PMeBr (11.82 g, 33.1 mmol) in THF (120 mL), *n*-BuLi (2 M) was added in cyclohexane (18 mL) dropwise at 0 °C under nitrogen. The reaction mixture was stirred at room temperature for 1 h. Then, 2-nitrobenzaldehyde (5 g, 33.1 mmol) was added and the mixture was heated to 65 °C for 5 h The reaction was quenched by the addition of saturated aqueous solution of NH_4_Cl. The aqueous phase was extracted with CH_2_Cl_2_ (3 × 20 mL). The combined organic layers were dried over Na_2_SO_4_ and evaporated under reduced pressure. The obtained residue was purified by column chromatography (silica gel, *n*-hexane/ethyl acetate 95:5) to afford the product (Yellow oil, 80% yield, 77% yield in reference [34]). ^1^H NMR (300 MHz, CD_2_Cl_2_) δ (ppm) 5.38 (dd, 1H, J = 10.5 Hz, J = 3 Hz), 5.67 (dd, 1H, J = 3 Hz, J = 18 Hz), 7.05 (dd, 1H, J = 10.5 Hz, J = 18 Hz), 7.3–7.36 (m, 1H), 7.48–7.58 (m, 2H), 7.80–7.82 (m, 1H). Spectroscopic data are in agreement with those reported in the literature [38].

#### 4.2.2. Synthesis of 2-vinylaniline (3a)

Adapted from reference [25]. To a solution of 2-nitrostyrene (3 g, 20 mmol) in ethanol (50 mL), iron powder (6.9 g, 124 mmol, 6.2 equiv) and concentrated HCl (2.33 mL, 24 mmol, 1.2 equiv) were added. After 3 h of reflux, the mixture was cooled to room temperature, and Na_2_CO_3_ was added by portions until gas evolution ceased. After filtration over celite, the filtrated was extracted with H_2_O and a saturated solution of NaCl. The organic layer was dried over Na_2_SO_4_, filtered, and concentrated in vacuo. The residual oil was purified by column chromatography (alumina, *n*-hexane/ethyl acetate 95:5) to produce the product (yellow oil, 92% yield, 92% yield in reference [34]). ^1^H NMR (300 MHz, CDCl_3_) δ (ppm) 3.52 (s, 2H), 5.20 (dd, 1H, J = 3 Hz, J = 12 Hz), 5.52 (dd, 1H, J = 3 Hz, J = 18 Hz) 6.54 (m, 1H), 6.60–6.70 (m, 2H), 6.94–7.00 (m, 1H), 7.16–7.19 (m, 1H). Spectroscopic data are in agreement with those reported in the literature [38].

#### 4.2.3. Synthesis of *N*-Protected-2-vinylaniline (3b–f)

To a solution of 2-vinyl aniline (2 mmol, 0.24 g) and pyridine (1.4 equiv) in ethyl acetate (25 mL), 1.5 equiv of the corresponding anhydride or chloride reagent at room temperature was added. After 2 h, the mixture was washed successively with water, a saturated solution of NaHCO_3,_ and a saturated aqueous solution of brine. The organic layer was dried over anhydrous Na_2_SO_4_. The compounds were purified by chromatography (silica gel, *n*-hexane/ethyl acetate) or recristallisation in hexane.

*N-Acetyl-2-vinyl-aniline* (**3b**), brown oil, 92% yield,^1^H NMR (300 MHz, CDCl_3_), δ (ppm) 2.11 (s, 3H), 5.32 (dd, 1H, J = 3 Hz, J = 12 Hz) 5.60 (d, 1H, J = 15 Hz), 6.72 (dd, 1H, J = 12 Hz, J = 18 Hz) 7.05–7.22 (m, 3H), 7.34–7.36 (m, 1H), 7.68–7.71 (m, 1H) ^13^C NMR (75 MHz, CDCl_3_) 24.3 (-CH_3_), 117.9 (=CH_2_), 124.0 (-CH=), 125.5 (CH_ar_), 126.8 (CH_ar_), 128.5 (CH_ar_), 130.6 (C_ar_), 132.3 (CH_ar_), 134.4 (C_ar_), 168.5 (-CO-) HRMS (ESI-MS) calculated for C_10_H_12_NO 162.0919 found 162.0921.

*N-FMoc-2-vinyl-aniline* (**3c**)*,* white solid, 82% yield, ^1^H NMR (300 MHz, CDCl_3_) δ (ppm) 4.20 (t, 1H, J = 6 Hz), 4.45 (d, 2H, J = 6 Hz), 5.35 (dd, 1H, J = 3 Hz, J = 12 Hz), 5.60 (dd, 1H, J = 3 Hz, J = 18 Hz), 6.48 (s, 1H), 6.73 (dd, J = 12 Hz, J = 18 Hz), 7.06–7.71 (m, 12H). ^13^C NMR (75 MHz, CDCl_3_) 47.2 (-CH-), 67.1 (-CH_2_-O), 118.1 (C_ar_), 120.1 (=CH_2_), 125.1 (-CH=), 127.0 (C_ar_), 127.1 (C_ar_), 127.8 (C_ar_), 128.6 (C_ar_), 132.1 (2 C_ar_), 134.3 (C_ar_), 141.4 (2 C_ar_), 143.8 (-CO-) HRMS (ESI-MS) calculated for C_23_H_20_NO_2_ 342.1494 found 342.1485.

*N-BOC-2-vinyl-aniline* (**3d**), white solid, 76% yield, ^1^H NMR (300 MHz, CDCl_3_) δ (ppm) 1.44 (s, 9H), 5.32 (dd, 1H, J = 3 Hz, J = 12Hz), 5.57 (dd, 1H, J = 3 Hz, J = 18 Hz), 6.35 (s, 1H), 6.73 (dd, 1H, J = 18 Hz, J = 12 Hz), 7.00–7.02 (m, 1H), 7.18 (m, 1H), 7.30 (m, 1H), 7.72 (m, 1H). ^13^C NMR (75 MHz, CDCl_3_) 27.3 (3 CH_3_-), 79.5 (-C-O), 116.8 (=CH_2_), 122.9 (-CH=), 125.9 (CH_ar_), 127.5 (CH_ar_), 128.2 (C_ar_), 131.2 (CH_ar_), 134.0 (C_ar_), 152.0 (-CO-) HRMS (ESI-MS) calculated for C_13_H_18_NO_2_ 220.1324 found 220.1328.

*N-mesyl(methanesulfony)l-2-vinyl-aniline* (**3e**), white solid, 92% yield, ^1^H NMR (300 MHz, CDCl_3_) δ (ppm) 2.92 (s, 3H), 5.40 (dd, 1H, J = 12 Hz, J = 3 Hz), 5.65 (dd, 1H, J = 18 Hz, J = 3 Hz), 6.48 (s, NH 1H), 6.87 (dd, 1H, J = 18 Hz, J = 12), 7.16–7.24 (m, 2H), 7.39–7.45 (m, 2H). Spectroscopic data are in agreement with those reported in the literature [39].

*N-p-tosyl(toluenesulfonyl)-2-vinyl-aniline* (**3f**), white solid, 89% yield, ^1^H NMR (300 MHz, CDCl_3_) δ (ppm) 2.31 (s, 3H), 5.18 (dd, 1H, J = 12 Hz, J = 3 Hz), 5.43 (dd, 1H, J = 18 Hz, J = 3 Hz), 6.47 (m, 2H), 7.07–7.18 (m, 4H), 7.23–7.28 (m, 2H), 7.52–7.55 (m, 2H). Spectroscopic data are in agreement with those reported in the literature [39].

#### 4.2.4. Synthesis of *N*-(2-Chlorophenyl)-2-vinyl-aniline (3g)

Adapted from reference [26]. The amounts of 0.025 mmol of BrettPhos, 0.0075 mmol of Pd_2_(dba)_3_, equiv of NaO*t*Bu, and 1.2 mmol of 2-chloroaniline were mixed in ovendried schlenk tube under nitrogen atmosphere. An amount of 1 mmol of 2-Bromostyrene in degassed dioxane was added under an argon atmosphere. The tube was then placed in a preheated oil bath at 110 °C, and mixture was stirred for 18 h. After being cooled down to room temperature, the solution was quenched with water and diluted with ethyl acetate. An organic layer was dried on Na_2_SO_4_ and filtered. After concentration under reduced pressure, the crude mixture was purified by column chromatography (silica gel, *n*-hexane/ethyl acetate 90:10). Yellow oil, 86% yield, ^1^H NMR (300 MHz, CDCl_3_), δ (ppm) 5.21 (dd, 1H, J = 3 Hz, J = 12 Hz), 5.63 (dd, 1H, J = 3 Hz, J = 18 Hz), 5.88 (s, NH 1H), 6.62–6.68 (m, 1H), 6.74–6.83 (m, 2H), 6.94–7.06 (m, 2H), 7.15–7.17 (m, 2H), 7.23–7.26 (m, 1H), 7.45–7.48 (m, 1H) ^13^C NMR (75 MHz, CDCl_3_) 115.2 (CH_ar_), 116.5 (CH_ar_), 119.8 (=CH_2_), 120.8 (C_ar_), 123.3 (-CH=), 124.5 (CH_ar_), 127.0 (CH_ar_), 127.6 (CH_ar_), 128.8 (CH_ar_), 129.6 (CH_ar_), 132.4 (C_ar_), 132.6 (CH_ar_), 138.5 (C_ar_), 141.4 (C_ar_), HRMS (ESI-MS) calculated for C_14_H_13_ClN 230.0720 found 230.0723.

*Synthesis of N-methyl-N-(2-chlorophenyl)-2-vinyl-aniline* (**8**). NaH (1.5 equiv) was washed 3 times with hexane (3 × 10 mL) under nitrogen atmosphere, and 20 mL of anhydrous THF was added, and the solution was cooled at 0 °C. Then, *N*-(2-Chlorophenyl)-2-vinyl-aniline (**3g**) was added and the conditions were kept for 1 h. An amount of 10 equiv of MeI was added to the mixture and the reaction was left for 24 h. The mixture was quenched with water-CH_2_Cl_2,_ and the organic phases were dried over Na_2_SO_4_. The product was purified by column chromatography (silica gel, *n*-hexane/ethyl acetate 90:10). Yellow oil, 77% yield, ^1^H NMR (300 MHz, CDCl_3_) δ (ppm) 3.07 (s, 3H), 5.05 (dd, 1H, J = 3 Hz, J = 12 Hz), 5.50 (dd, 1H, J = 3 Hz, J = 18 Hz), 6.78–6.81 (m, 1H), 6.87–7.02 (m, 4H), 7.07–7.12 (m, 2H), 7.21–7.25 (m, 1H), 7.40–7.43 (m, 1H) ^13^C NMR (75 MHz, CDCl_3_) 41.8 (-CH_3_), 114.1 (=CH_2_), 122.3 (-CH=), 123.4 (CH_ar_), 123.7 (CH_ar_), 124.0 (CH_ar_), 127.1 (CH_ar_), 127.4 (CH_ar_), 128.4 (CH_ar_), 128.9 (C_ar_), 130.9 (CH_ar_), 133.4 (C_ar_), 134.3 (CH_ar_), 148.3 (C_ar_), 148.6 (C_ar_**),** HRMS (ESI-MS) calculated for C_15_H_15_ Cl N 244.0810 found 244.0819.

### 4.3. Interrupted Hydroaminomethylation Procedure

To a solution of *N*-protected-2-vinyl-aniline (0.6 mmol in 20mL toluene), 0.4 mol% of ligand and 0.2mol% of Rh(acac)(CO)_2_ were added. The mixture was placed in a TOP Industries reactor, under 20 bar syngas pressure at a temperature of 60 °C. Once the temperature was reached, the pressure increased to 30 bar and stirring was setting at 1000 rpm. The reaction was monitored by GC and stopped until no detected starting material was present. Once the reaction is complete, the reactor was cooled and the system is carefully depressurized. The reaction was concentrated by evaporating the solvent. The products were separated and purified by column chromatography (alumina, *n*-hexane/ethyl acetate 70:30) 

*3-methyl-N-H-indole* (**7a**) (skatole, marketed compound)**,** White solid, >99% Yield ^1^H NMR (300 MHz, CDCl_3_) δ (ppm) 2.26 (d, 3H, J = 1 Hz),6.89 (m, 1H, J = 1 Hz), 7.02–7.14 (m, 2H), 7.25–7.28 (m, 1H), 7.49–7.52 (m, 1H), 7.79 (s, 1H). Spectroscopic data are in agreement with those reported in the literature.

*3-methyl-N-acetyl-indole* (**7b**) (N-acetyl skatole, marketed compound), white solid, 23% yield ^1^H NMR (300 MHz, CD_2_Cl_2_) δ (ppm) 2.10 (s, 3H), 2.57 (s, 3H), 7.02–7.07 (m, 1H), 7.47–7.48 (m, 1H), 7.83–7.86 (m, 1H), 8.60–8.63 (m, 1H), 11.57 (s, 1H) as reported in the literature.

*3-methyl-N-Fmoc-indole* (**7c**)*,* white solid, (mixture of 3-methyl-*N*-Fmoc-indole, skatole and *9*-methylen-9*H*-fluorene) ^1^H NMR (300 MHz, CDCl_3_) δ (ppm) 2.11 (s, CH_3_ 3H, N-Fmoc skatole), 2.29 (d, CH_3_ 3H skatole), 3.85 (d, J = 6.5 Hz, 1H CH, N-Fmoc skatole), 4.52 (t, CH 1H), 5.23 (s, 2H, CH_2_ 9-methylene-9H-fluorene), 6.03 (s, 1H CH_Ar_ N-Fmoc skatole), 6.88 (s, 1H CH_Ar_ skatole), 7.19–7.03 (m, 2H Ar), 7.43–7.22 (m, 6H, Ar), 7.54 (d, J = 7.2 Hz, Ar, 2H), 7.77–7.61 (m, Ar, 4H), 7.86 (s, 1H, NH skatole). ^13^C NMR (76 MHz, CDCl_3_) 9.73, 30.97, 46.24, 46.41, 46.99, 47.54, 49.40, 50.43, 53.50, 65.21, 67.00, 71.69, 73.53, 107.88, 111.05, 111.07, 111.64, 118.84, 119.11, 119.14, 119.82, 120.00, 120.10, 120.13, 120.27, 120.32, 120.51, 121.08, 121.69, 121.86, 124.80, 124.98, 125.02, 125.12, 125.17, 127.08, 127.14, 127.26, 127.41, 127.45, 127.65, 127.67, 128.10, 128.22, 128.32, 128.57, 128.82, 136.33, 138.09, 140.21, 141.26, 141.40, 142.75, 143.41, 144.15, 144.31, 179.12.

*3-methyl-N-BOC-indole* (**7d**)*,* white solid, 15% yield, ^1^H NMR (300 MHz, CDCl_3_) δ 1.65 (s, CH_3_ 9H), 2.26 (s, CH_3_ 3H), 7.15–7.40 (m, Ar 3H), 7.44–7.54 (m, Ar 1H), 8.11 (d, J = 8.2 Hz, 1H). in accordance with the reference [40].

*3-methyl-N-mesyl-indole* (**7e**), white solid, >99% yield (36% yield in reference [38]), ^1^H NMR (300 MHz, CD_2_Cl_2_) 2.21 (d, 3H, 3 Hz), 2.93 (s, 3H), 7.11 (m, 1H, J = 3 Hz), 7.20–7.30 (m, 1H), 7.47–7.50 (m, 1H), 7.77–7.80 (m, 1H) as reported in reference [41].

*3-methyl-N-tosyl-indole* (**7f**), white solid, >99% yield (67% yield in reference [38]), ^1^H NMR (300 MHz, CD_2_Cl_2_) δ (ppm) 2.15 (d, 3H, J = 3 Hz), 2.24 (d, 3H, J = 1 Hz), 7.13–7.23 (m, 5H), 7.36–7.39 (m, 1H), 7.63–7.66 (AA’BB’, 2H), 7.87–7.88 (m, 1H) as reported in reference [41].

*3-methyl-N-(2-chlorophenyl)-indole* (**7g**), yellow oil, >99% yield, ^1^H NMR (300 MHz, CDCl_3_) δ (ppm) 2.32 (s, CH_3_ 3H), 6.95 (s, C=CH, 1H), 7.02–7.57 (m, 8H) ^13^C NMR (75 MHz, CDCl_3_) 9.8 (CH_3_-), 110.6 (CH_ar_), 112.6 (-C=), 119.15 (CH_ar_), 119.8 (CH_ar_), 122.3 (CH_ar_), 126.3 (CH_ar_), 127.7 (CH_ar_), 128.7 (C_ar_), 129.4 (CH_ar_), 130.9 (C_ar_), 137.0 (C_ar_), 137.2 (C_ar_), HRMS (ESI-MS) Calculated for C_15_H_12_NCl 241.0651 found 241.0658

*3-methyl-N-acetyl-indoline-2-ol,* (**6b**) ^1^H NMR (300 MHz, CD_2_Cl_2_) (the numbering of the carbon atoms follows that of the indoline unit in Figure 2) δ (ppm) 1.20 (d, C_31_H_3_, J = 3 Hz, 3H), 2.10 (s, H_3_C-CO, 3H), 3.11–3 (m, HC_3_, 1H), 3.30 (d, J = 3 Hz, -OH, 1H), 5.35 (dd, -C_2_H-OH, J = 6 Hz, J = 3 Hz, 1H), 6.91–7.67 (m, HC_4_-C_7_H, 4H). ^13^C NMR (75 MHz, CDCl_3_) 19.1, 31.5, 43.1, 93.1, 125.5, 126.8, 128.5, 130.6, 132.3, 134.4, 169, HRMS (ESI-MS) Calculated for C_11_H_13_NO_2_ 192.2303 found 192.1029

*3-methyl-N-Fmoc-indoline-2-ol* (**6c**), ^1^H NMR (300 MHz, CDCl_3_) (the numbering of the carbon atoms follows that of the indoline unit in Figure 2) δ (ppm). 1.25 (d, C_31_H_3_, J = 5.1 Hz, 3H), 3.11–2.86 (m, C_3_H, 1H), 4.76–4.70 (m, C_2_H, 1H), 5.43–5.46 (m, CH_2_O), 6.88 (d, C_2_H-OH J = 6.7 Hz, 1H), 7.00–7.46 (m, C_4_H-C_7_H, 4H), 7.54–7.73 (CH_Ar_, 8H). ^13^C NMR (75 MHz, CDCl3) δ 19.3, 42.9, 47.0, 68.5, 80.5, 114.4, 119.0, 120.2, 123.2, 124.6, 124.9, 127.4, 127.4, 127.8, 135.1, 141.5, 143.6, 162.8.

*3-methyl-N-mesylindoline-2-ol* (**6e**), white solid, >99% Yield, diastereomer *trans* ^1^H NMR (300 MHz, CDCl_3_) (the numbering of the carbon atoms follows that of the indoline unit in Figure 2) δ (ppm) 1.22 (d, C_31_H_3_ J = 3 Hz, 3H), 2.94 (s, S-CH_3_, 3H), 3.12 (qd, C_3_H, J_HC-CH3_ = 6 Hz, J_HC-CH_ = 3 Hz, 1H), 3.58 (d, -OH, J = 3 Hz, 1H), 5.37 (dd, C_2_H, J = 6 Hz, J = 3 Hz, 1H), 6.96–7.23 (m, C_4_H-C_7_H, 4H) diasteromer *cis* ^1^H NMR (300 MHz, CDCl_3_) δ (ppm) 1.29 (d, C_31b_H_3_ J = 3 Hz, 3H), 2.94 (s, S-CH_3_, 3H), 3.39 (m, C_3b_H_,_ 1H), 3.33 (d, -OH, J = 3 Hz, 1H), 5.71 (t, C_2_H, J = 6 Hz, 1H), 6.96–7.23 (m, C_4_H-C_7_H, 4H) ^13^C NMR (75 MHz, CDCl_3_) 19.14, 38.88, 44.11, 93.24, 112.64, 123.82, 124.79, 128.38, 133.69, 139.25 HRMS (ESI-MS) Calculated for C_10_H_13_NO_3_S 227.0615 found 227.0616. 

*3-methyl-N-tosylindoline-2-ol* (**6f**), white solid, >99% Yield, diastereomer *cis*, ^1^H NMR (300 MHz, CDCl_3_) (the numbering of the carbon atoms follows that of Figure 2) δ (ppm) 0.85 (d, C_31b_H_3_ J = 6 Hz, 3H), 2.28 (s, C_17_H_3_, 3H), 3.01 (qd, C_3b_H_,_ J_HC-CH3_ = 6 Hz, J_HC-CH_=3 Hz, 1H), 3.40 (d, -OH, J = 3 Hz, 1H), 5.31 (dd, C_2_H, J = 3 Hz, J = 3 Hz, 1H), 6.91–7.67 (m, C_4_H-C_7_H and C_11_H-C_16_H, 8H); diastereomer *trans ^1^H* (300 MHz, CDCl_3_) δ (ppm) 1.21 (d, C_31_H_3_ J = 3 Hz, 3H), 2.03 (s, C_17_H_3_, 3H,), 3.12–3.19 (m, C_3_H, 1H), 3.32 (d, J = 3 Hz, -OH, 1H) 5.31 (m, C_2_H, 1H), 6.91–7.67 (m, C_4_H-C_7_H and C_11_H-C_16_H, 8H); ^13^C NMR (75 MHz, CDCl_3_) 19.0, 21.3, 43.9, 93.1, 113.9, 123.9, 124.5, 127.0, 128.0, 129.8, 134.4, 135.3, 139.2, 144.7. HRMS (ESI-MS) Calculated for C_16_H_17_NO_3_S 303.0925 found 303.0929.

## Figures and Tables

**Table 1:** Interrupted intramolecular HAM reaction of 2-vinylanilines **3**.

Entry	3	pKa ^a^	% yield ^b^ of 6 (dr ^c^)	% yield ^b^ of 7
1	**3a** (R=H)	-	0	>99
2	**3g** (R=2-ClPh)	-	0	>99
3	**3b** (R=COCH_3_)	4.74	77 ^d^ (5:1)	23
4	**3c** (R=Fmoc)	6.32	85 ^d^ (4.5:1)	15
5	**3d** (R=BOC)	6.33	85 ^d^ (4.5:1)	15
6	**3e** (R=Ms)	−1.61	>99 (4.5:1)	0
7	**3f** (R=Ts)	−2.14	>99 (5:1)	0

^a^ Predicted pKa for the corresponding organic acids using chemicalize software https://chemicalize.com/ (accessed 22 september 2021). ^b^ Yields obtained after purification on column chromatography using neutral alumina. ^c^ dr: diastereomeric ratio determined by CPG. ^d^ This product is unstable and readily decomposes to 3-methyl-*N*-H-indole (skatole).

**Table 2:** Effect of chiral ligands on the intramolecular interrupted HAM reaction using (**3f**) as a model substrate.

Entry	Ligand	Conversion	d.r ^b^	ee (%) ^a^
1	PPh_3_	99	5:1	-
2	**L1**	95	2.5:1	21
3	**L2**	98	3:1	21
4	**L3**	95	2:1	22
5	**L4**	99	3:1	18
6	**L5**	95	2:1	19
7	**L6**	98	10:1	43
8	**L7**	97	3:1	23

^a^ Determined by chiral HPLC for the trans diastereomer. ^b^ Determined by GC.

## Data Availability

Not applicable.

## Supplementary Materials

The following are available online, S1. Hydroformylation of *N*-(2-chlorophenyl)-2-vinyl aniline, S2. Molecular view of compound 7a with the atom labelling scheme, S3. Tables of X-ray structural analysis of compound 7a and 3-methyl-*N*-tosyl-indolyl-2-ol (6f), S4. ^1^H and ^13^C NMR Spectra of **3c**, S5. ^1^H and ^13^C NMR Spectra of **3d**, S6. ^1^H and ^13^C NMR Spectra of **3e**, S7. ^1^H and ^13^C NMR Spectra of **3g**, S8. ^1^H and ^13^C NMR Spectra of **7g**, S9. ^1^H and ^13^C NMR Spectra of **7a**, S10. ^1^H and ^13^C NMR Spectra of **7b**, S11. ^1^H and ^13^C NMR Spectra of **6e**, S12. ^1^H and ^13^C NMR Spectra of **6f**.

## Sample Availability

Samples of the compounds are available from the authors.
