# Supplementary material for: Interrupted Intramolecular Hydroaminomethylation of N-Protected-2-vinyl Anilines: Novel Access to 3-Substitued Indoles or Indoline-2-ols
Source: Molecules. 2022 Feb 5;27(3):1074. doi: 10.3390/molecules27031074 (PMC8840357; doi:10.3390/molecules27031074)

Supporting Information

Interrupted Intramolecular Hydroaminomethylation of *N*-Protected-2-vinyl anilines: Novel access to 3-substitued indoles or indoline-2-ols.

Frank Hochberger-Roa [1], Perla H. García-Ríos [1,2], José G. López-Cortés [2], M. Carmen Ortega-Alfaro [3], Jean-Claude Daran[1], Maryse Gouygou [1], Martine Urrutigoïty [1*]

[1] Laboratoire de Chimie de Coordination (LCC), Université de Toulouse, CNRS, 31030 Toulouse, France; jeanclaude-daran@lcc-toulouse.fr; gouygou@lcc-touloouse.fr, martine.urrutigoity@ensiacet.fr

[2] Instituto de Química, Universidad Nacional Autónoma de México, Circuito Exterior, Ciudad Universitaria, Coyoacán C.P. 04510, CdMx, México; jglcvw@unam.mx

[3] Instituto de Ciencias Nucleares, Universidad Nacional Autónoma de México, Circuito Exterior, Ciudad Universitaria, Coyoacán C.P. 04510, CdMx, México; carmen.ortega@correo.nuclares.unam.mx

Table of contents

S1 Hydroformylation of N-(2-chlorophenyl)-2-vinyl aniline

S2 Figure1: Molecular view of compound **7a**

S3 Tables of X-ray structural analysis of **7a** and **6f** (3-methyl-N-tosyl-indolyl-2-ol)

S4 ^{1}H and ^{13}C NMR Spectra of **3c** (N-Fmoc-2-vinyl aniline)

S5 ^{1}H and ^{13}C NMR Spectra of **3d** (N-acetyl-2-vinyl aniline)

S6 ^{1}H and ^{13}C NMR Spectra of **3e** (N-mesyl-2-vinyl aniline)

S7 ^{1}H and ^{13}C NMR Spectra of **3g** (N-(2-chlorophenyl)-2-vinyl aniline)

S8 ^{1}H and ^{13}C NMR Spectra of **7g** (3-methyl-N-(2-chlorophenyl)-indole)

S9 ^{1}H and ^{13}C NMR Spectra of **7a** {2-[2-N-(2-chlorophenyl)methylamino)phenyl]propanal}

S10 ^{1}H and ^{13}C NMR Spectra of **7b** {3-[2-N-(2-chlorophenyl)methylamino)phenyl]propanal}

S11 ^{1}H and ^{13}C NMR Spectra of **6e** (3-methyl-N-mesyl-indoline-2-ol)

S12 ^{1}H and ^{13}C NMR Spectra of **6f** (3-methyl-N-tosyl-indoline-2-ol)

S1.Hydroformylation of N (2-chlorophenyl)-2-vinyl aniline

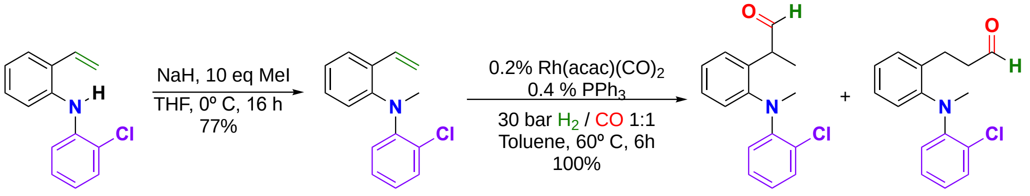

4 **4'** **7a** **7b**

To a solution of *N*-protected-2-vinyl-aniline (0.6mmole) in toluene (20 mL) was added $Rh(acac)(CO)_2$ (0.001 mmol, 0.2%) and PPh_3 (0.002 mmol, 0.4%). The mixture was transferred into the autoclave 90 mL inox (TOP INDUSTRIE), which was flushed several times with H2/CO gas, and the mixture was stirred at 200 rpm. The autoclave was pressurized to an initial 20 bar pressure of syngas (H_2/CO_2 1:1), heated to the 60°C. Once the temperature is reached, the pressure is increased to 30 bar and the stirring was setting at 1000 rpm. The reaction was monitored by GC and stopped until no detected starting material. After the catalytic reaction, the autoclave was then cooled and carefully depressurized. The conversion and selectivity of the reaction was determined by GC. The mixture was evaporated and analyzed by NMR. The two aldehydes were separated by chromatography (silica gel, n- hexane/ethyl acetate 95/5)

2-[2-N-(2-chlorophenyl)methylamino)phenyl]propanal (7a): yellow solid, ^{1}H (300 MHz, $CDCl_3$) δ (ppm) 1.12 (d, J=4.5 Hz, 3H), 3.11 (s, 3H), 4.01 (cuat, 1H, J=6 Hz), 6.86-7.00 (m, 3H), 7.06-7.11 (m, 3H), 7.18-7.29 (m, 3H), 9.37 (s, 1H). ^{13}C (75 MHz, $CDCl_3$) 14.5 (C-CH_3), 42.3 (N-CH_3), 46.9 (-CH-CO), 123.5 (CH_{ar}), 124.3 (CH_{ar}), 124.5(CH_{ar}), 125.1 (CH_{ar}), 127.7 (CH_{ar}), 128.3 (CH_{ar}), 128.9(C_{ar}), 129.5 (CH_{ar}), 131.2 (CH_{ar}), 134.2 (C_{ar}), 148.5 (C_{ar}), 149.6 (C_{ar}), 201.5 (COH).

3-[2-N-(2-chlorophenyl)(methylamino)phenyl]propanal (7b): brown oil, ^{1}H (300 MHz, $CDCl_3$) 2.52-2.56 (m, 2H), 2.60-2.65 (m, 2H), 3.12 (s, 3H), 6.76-6.79 (m, 1H), 6.88-6.93 (m, 1H), 6.99-7.16 (m, 5H), 7.27-7.30 (m, 1H), 9.61 (T, 1H, J=3 Hz).^{13}C (75 MHz, $CDCl_3$) 23.1 (Ar-CH_2-), 28.7 (-CH_2-CO), 40.4 (N-CH_3), 42.5(CH_{ar}), 121.3(CH_{ar}), 123.2(CH_{ar}), 123.3(CH_{ar}), 123.5(CH_{ar}), 126.2(CH_{ar}), 126.6(CH_{ar}), 128.1(C_{ar}), 129.3(CH_{ar}), 130.0(CH_{ar}), 135.0(C_{ar}), 147.4(C_{ar}), 147.6(C_{ar}), 201.2 (COH).

S2 Figure S1: Molecular view of compound **7a** with the atom labelling scheme. Ellipsoids are drawn at the 30% probability level. H atoms are represented as small circle of arbitrary radii.

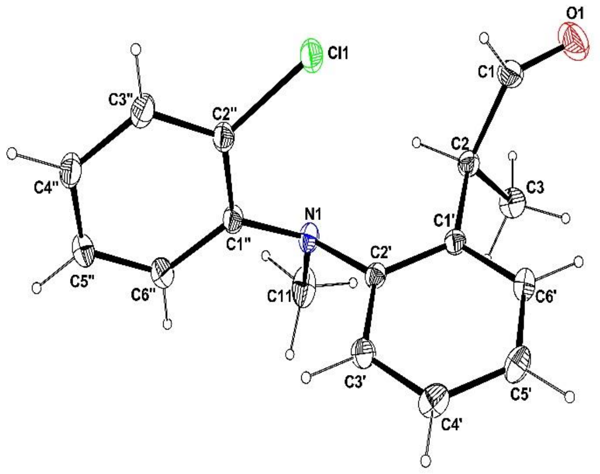

S3 Tables of X-ray structural analysis of compound **7a** and 3-methyl-N-tosyl-indolyl-2-ol (**6f**)

Single crystal of each compound was mounted under inert perfluoropolyether at the tip of glass fiber and cooled in the cryostream of a Nonius APEXII CCD diffractometer.
The structures were solved by direct methods SHELX[1] and refined by least-squares procedures on F^2 using SHELXL-2018[2]. All H atoms attached to carbon were introduced in calculation in idealised positions and treated as riding models, only the methine H attached to C2 in 3-methyl-N-tosyl-indoline-2-ol (**5R)** has been refined. The hydroxyl H in 5R has been also treated as riding on the parent O atom. In compound **5R**, within the N1 C2 C3 C3A C7A ring the C3 atom bearing the methyl group is disordered over two position in the ratio 70/30, resulting in the inversion of configuration on C3 (C3b). The disordered model has been refined using the tools available in SHELXL-2018. The drawing of the molecules was realised with the help of ORTEP32[3,4]. Crystal data and refinement parameters are shown in Table 1.

Table S1. Crystal data and structure refinement

Identification code	**7a**	**6f**
Empirical formula	$C_{16} H_{16} Cl N O$	$C_{16} H_{17} N O_3 S$
Formula weight	273.75	303.36
Temperature, K	180(2)	180(2)
Wavelength, Å	0.71073	0.71073
Crystal system	Monoclinic	Orthorhombic
Space group	$P2_1/n$	Pb a
a, Å	10.7681(6)	13.542(2)
b, Å	11.5110(5)	13.242(3)
c, Å	11.6060(7)	16.590(4)
α◎◎◎	90.0	90.0
◎◎◎◎	105.238(2)	90.0
γ◎◎◎	90.0	90.0
Volume, $Å^3$	1388.01(13)	2975.1(11)
Z	4	8
Density (calc), Mg/m^3	1.315	1.355

[1] Sheldrick, G.M., Crystal structure refinement with *SHELXL*. *Acta Cryst*. 2015, *C71*, 3-8. DOI 10.1107/S2053229614024218
[2] Sheldrick, G.M., *SHELXT* – Integrated space-group and crystal-structure determination. *Acta Cryst*. 2015, *A71*, 3-8. DOI 10.1107/S2053273314026370
[3] Farrugia, L. J., *ORTEP*-3 for Windows - a version of *ORTEP*-III with a Graphical User Interface *J. Appl. Cryst.* **1997**, 30, 565, DOI 10.1107/S0021889897003117
[4] Burnett, M. N., Johnson, C. K., *ORTEPIII*. Report ORNL-6895, Oak Ridge National Laboratory, Tennessee, USA, **1996**.

Abs. coefficient, mm^{-1}	0.266	0.227
F(000)	576	1280
Crystal size, mm^3	0.500 x 0.500 x 0.350	0.25 x 0.10 x 0.02
Theta range, °	2.297 to 28.276°.	2.880 to 24.107°.
Reflections collected	20972	9604
Indpt reflections (R_{int})	3434 (0.0333)	2364 (0.0778)
Absorption correction	Multi-scan	Multi-scan
Max. / min. transmission	0.7470 / 0.7024	0.7454 / 0.5482
Refinement method	F^2	F^2
Data /restraints/parameters	3435 / 0 / 175	2622 / 8 / 203
Goodness-of-fit on F^2	1.089	1.038
R1, wR2 [I>2σ(I)]	0.0413, 0.1150	0.0589, 0.1434
R1, wR2 (all data)	0.0484, 0.1268	0.1081, 0.1747
Residual density, e.Å^{-3}	0.636 / -0.504	0.485 / -0.364

Table S2. Bond lengths [Å] and angles [°].

**7a**			
Cl(1)-C(2'')	1.7384(14)	O(1)-C(1)	1.200(2)
N(1)-C(1'')	1.4163(17)	N(1)-C(2')	1.4405(16)
N(1)-C(11)	1.4637(19)	C(2')-C(3')	1.3943(18)
C(2')-C(1')	1.4015(17)	C(1'')-C(6'')	1.3991(19)
C(3')-C(4')	1.388(2)	C(2'')-C(3'')	1.3906(19)
C(4')-C(5')	1.385(2)	C(3'')-C(4'')	1.385(2)
C(5')-C(6')	1.386(2)	C(4'')-C(5'')	1.374(2)
C(6')-C(1')	1.3943(18)	C(5'')-C(6'')	1.390(2)
C(1')-C(2)	1.5175(18)	C(2)-C(1)	1.507(2)
C(1'')-C(2'')	1.398(2)	C(2)-C(3)	1.5275(19)

C(1'')-N(1)-C(2')	116.57(10)	C(3')-C(2')-C(1')	119.53(12)
C(1'')-N(1)-C(11)	116.03(11)	C(3')-C(2')-N(1)	120.58(11)
C(2')-N(1)-C(11)	112.67(11)	C(1')-C(2')-N(1)	119.82(11)
C(4')-C(3')-C(2')	120.97(13)	C(3'')-C(2'')-Cl(1)	118.01(11)
C(5')-C(4')-C(3')	119.84(13)	C(1'')-C(2'')-Cl(1)	120.10(10)
C(6')-C(5')-C(4')	119.33(13)	C(4'')-C(3'')-C(2'')	120.07(14)

C(5')-C(6')-C(1')	121.80(13)	C(5")-C(4")-C(3")	119.26(14)
C(6')-C(1')-C(2')	118.53(12)	C(4")-C(5")-C(6")	120.64(14)
C(6')-C(1')-C(2)	119.07(12)	C(5")-C(6")-C(1")	121.55(15)
C(2')-C(1')-C(2)	122.38(11)	C(1)-C(2)-C(1')	108.20(11)
C(2")-C(1")-C(6")	116.61(13)	C(1)-C(2)-C(3)	111.93(12)
C(2")-C(1")-N(1)	120.34(12)	C(1')-C(2)-C(3)	113.17(11)
C(6")-C(1")-N(1)	122.98(13)	O(1)-C(1)-C(2)	125.57(15)
C(3")-C(2")-C(1")	121.85(13)		

**6f**			
S(1)-O(11)	1.434(3)	S(1)-O(12)	1.422(3)
S(1)-N(1)	1.637(3)	S(1)-C(11)	1.758(3)
O(2)-C(2)	1.381(5)	N(1)-C(7A)	1.442(4)
N(1)-C(2)	1.499(5)	C(2)-C(3B)	1.543(11)
C(2)-C(3)	1.606(6)	C(3A)-C(3B)	1.610(12)
C(3)-C(3A)	1.503(6)	C(3B)-C(31B)	1.499(16)
C(3)-C(31)	1.529(7)	C(11)-C(12)	1.386(5)
C(3A)-C(7A)	1.374(5)	C(11)-C(16)	1.387(4)
C(3A)-C(4)	1.383(5)	C(12)-C(13)	1.386(5)
C(7A)-C(7)	1.381(5)	C(13)-C(14)	1.383(4)
C(4)-C(5)	1.361(6)	C(14)-C(15)	1.392(5)
C(5)-C(6)	1.363(6)	C(14)-C(17)	1.507(4)
C(6)-C(7)	1.401(6)	C(15)-C(16)	1.378(5)

O(12)-S(1)-O(11)	119.85(16)	O(12)-S(1)-C(11)	108.84(18)
O(12)-S(1)-N(1)	107.60(15)	O(11)-S(1)-C(11)	107.76(16)
O(11)-S(1)-N(1)	104.89(17)	N(1)-S(1)-C(11)	107.23(15)
C(2)-N(1)-S(1)	118.9(2)	C(7A)-N(1)-S(1)	121.8(2)
C(7A)-N(1)-C(2)	108.2(3)	C(31B)-C(3B)-C(2)	106.9(10)
O(2)-C(2)-N(1)	111.2(3)	C(31B)-C(3B)-C(3A)	112.0(10)
O(2)-C(2)-C(3B)	127.0(6)	C(2)-C(3B)-C(3A)	99.2(6)
N(1)-C(2)-C(3B)	99.8(5)	C(5)-C(4)-C(3A)	120.2(4)
O(2)-C(2)-C(3)	101.2(4)	C(4)-C(5)-C(6)	120.5(4)
N(1)-C(2)-C(3)	106.3(3)	C(5)-C(6)-C(7)	121.1(4)

C(3A)-C(3)-C(31)	110.0(5)	C(7A)-C(7)-C(6)	117.3(4)
C(3A)-C(3)-C(2)	101.1(4)	C(12)-C(11)-C(16)	120.2(3)
C(31)-C(3)-C(2)	109.2(5)	C(12)-C(11)-S(1)	119.6(3)
C(7A)-C(3A)-C(4)	119.2(4)	C(16)-C(11)-S(1)	120.1(3)
C(7A)-C(3A)-C(3)	113.5(3)	C(11)-C(12)-C(13)	119.5(3)
C(4)-C(3A)-C(3)	126.7(4)	C(14)-C(13)-C(12)	121.2(3)
C(7A)-C(3A)-C(3B)	103.6(5)	C(13)-C(14)-C(15)	118.0(3)
C(4)-C(3A)-C(3B)	133.6(5)	C(13)-C(14)-C(17)	121.3(3)
C(3A)-C(7A)-C(7)	121.7(3)	C(15)-C(14)-C(17)	120.6(3)
C(3A)-C(7A)-N(1)	110.5(3)	C(16)-C(15)-C(14)	121.7(3)
C(7)-C(7A)-N(1)	127.6(3)	C(15)-C(16)-C(11)	119.2(3)

S4 ^{1}H and ^{13}C NMR Spectra of **3c**

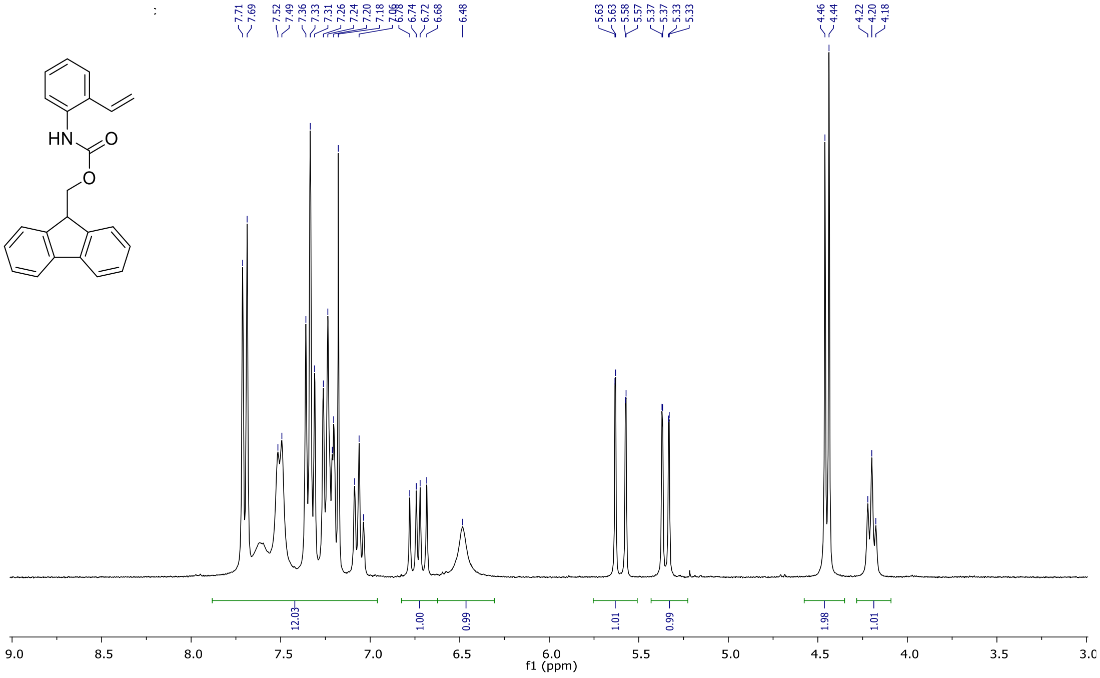

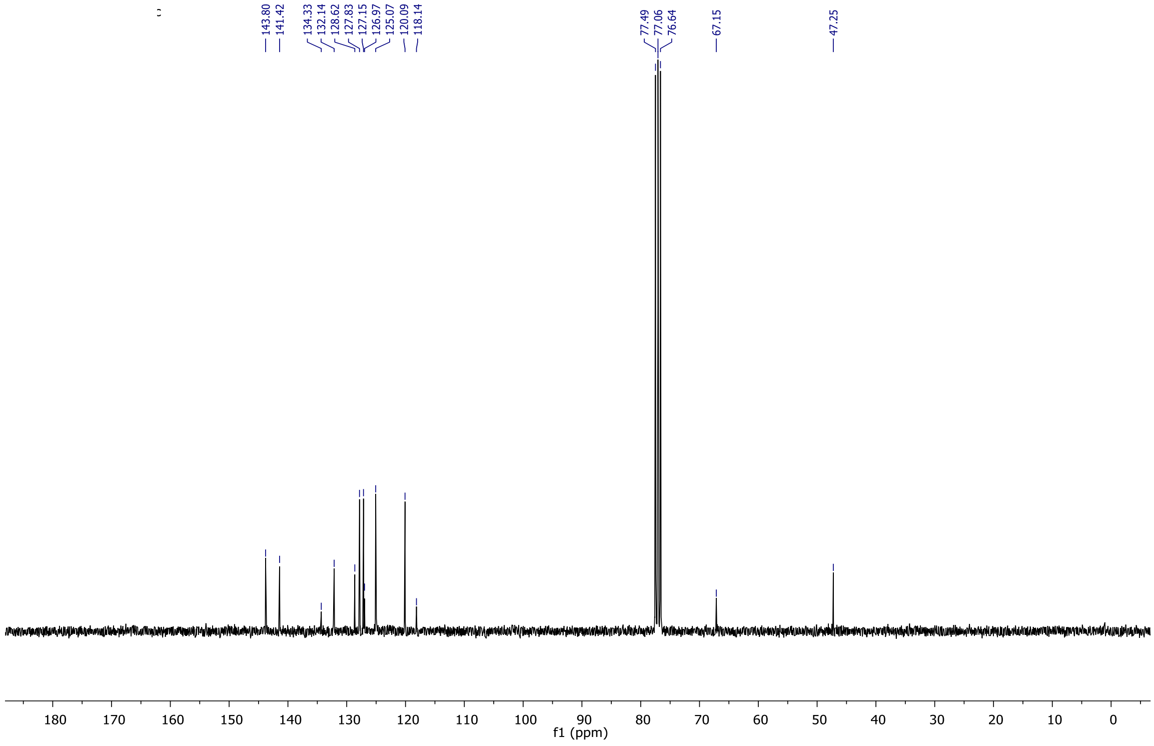

S5 ^{1}H and ^{13}C NMR Spectra of **3d**

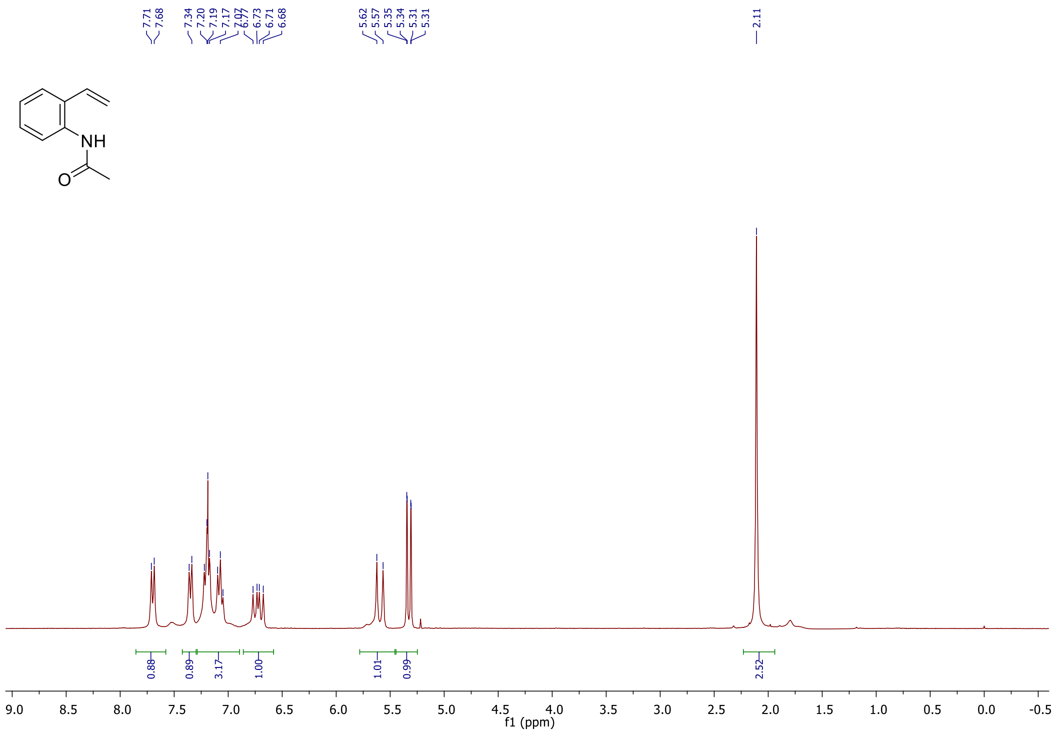

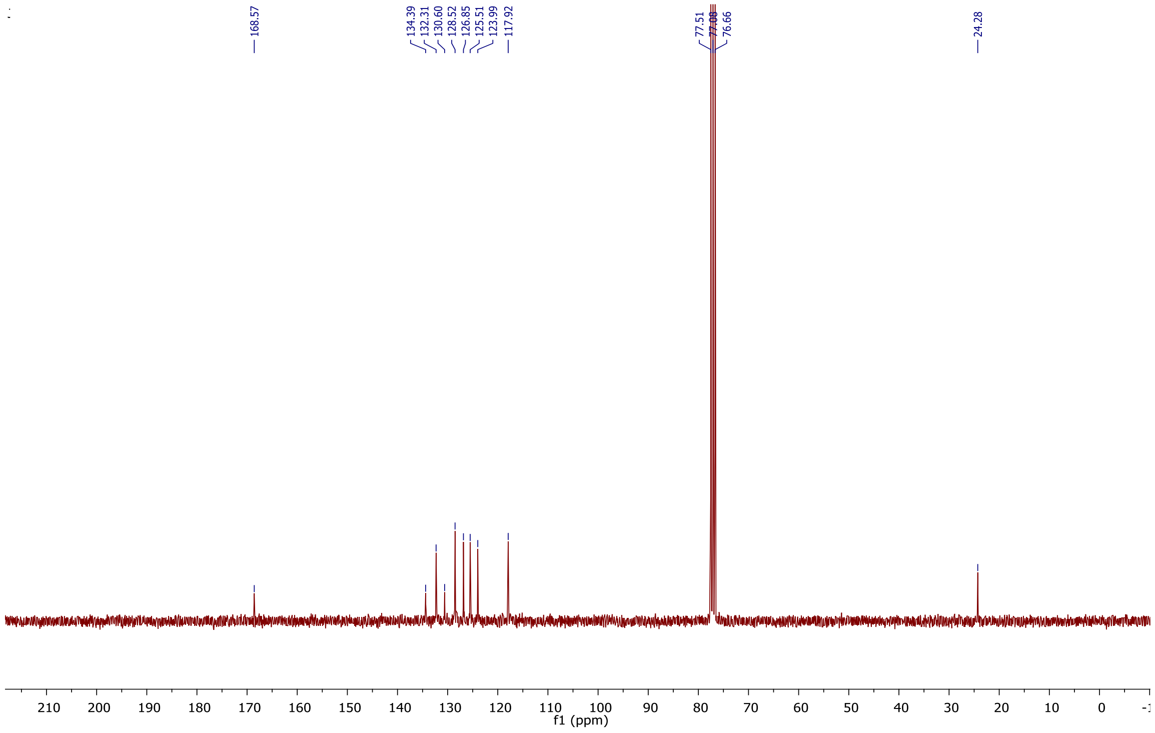

S6 ^{1}H and ^{13}C NMR Spectra of **3e**

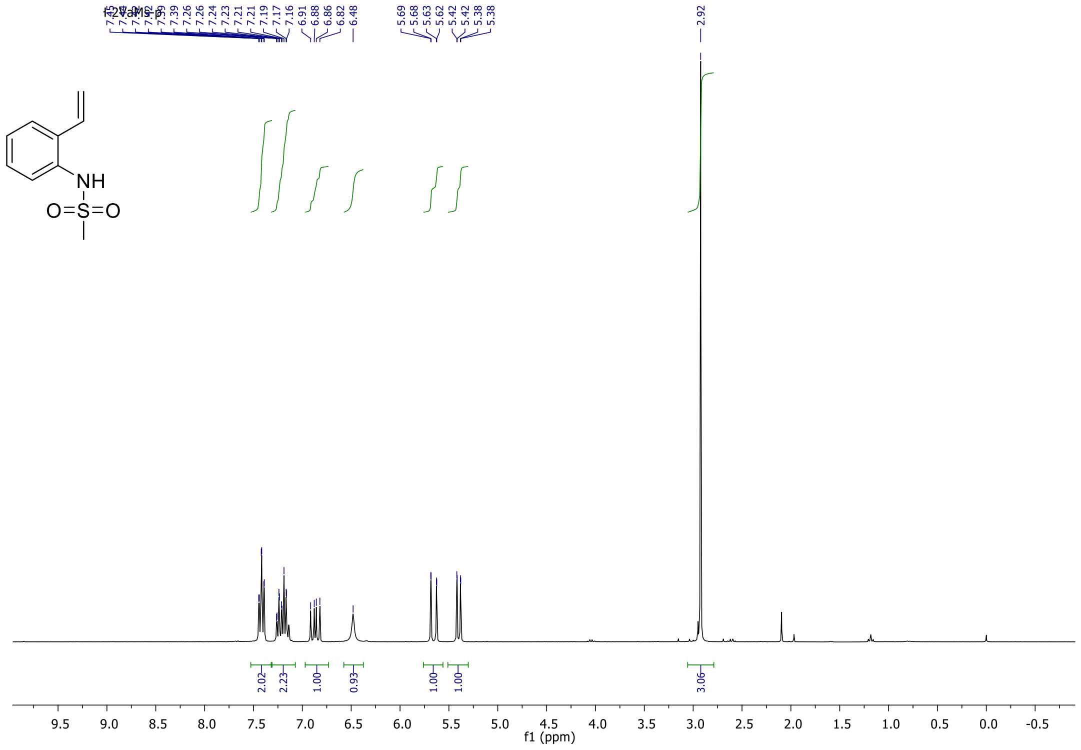

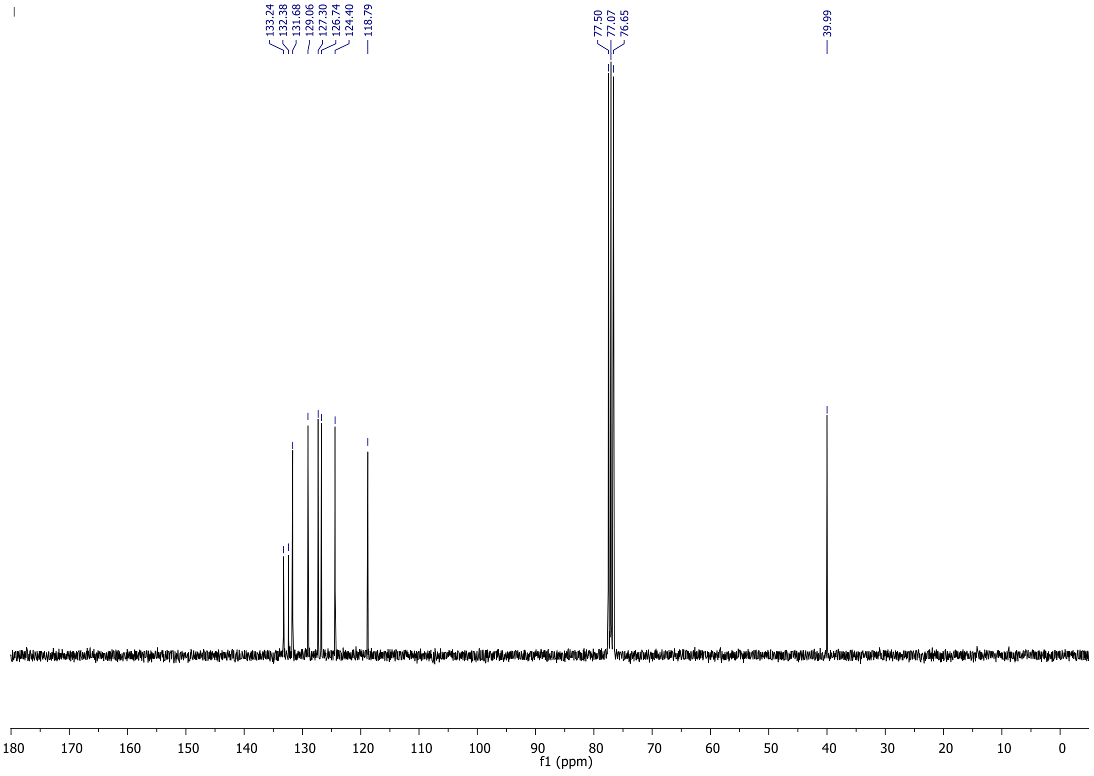

S7 ^{1}H and ^{13}C NMR Spectra of **3g**

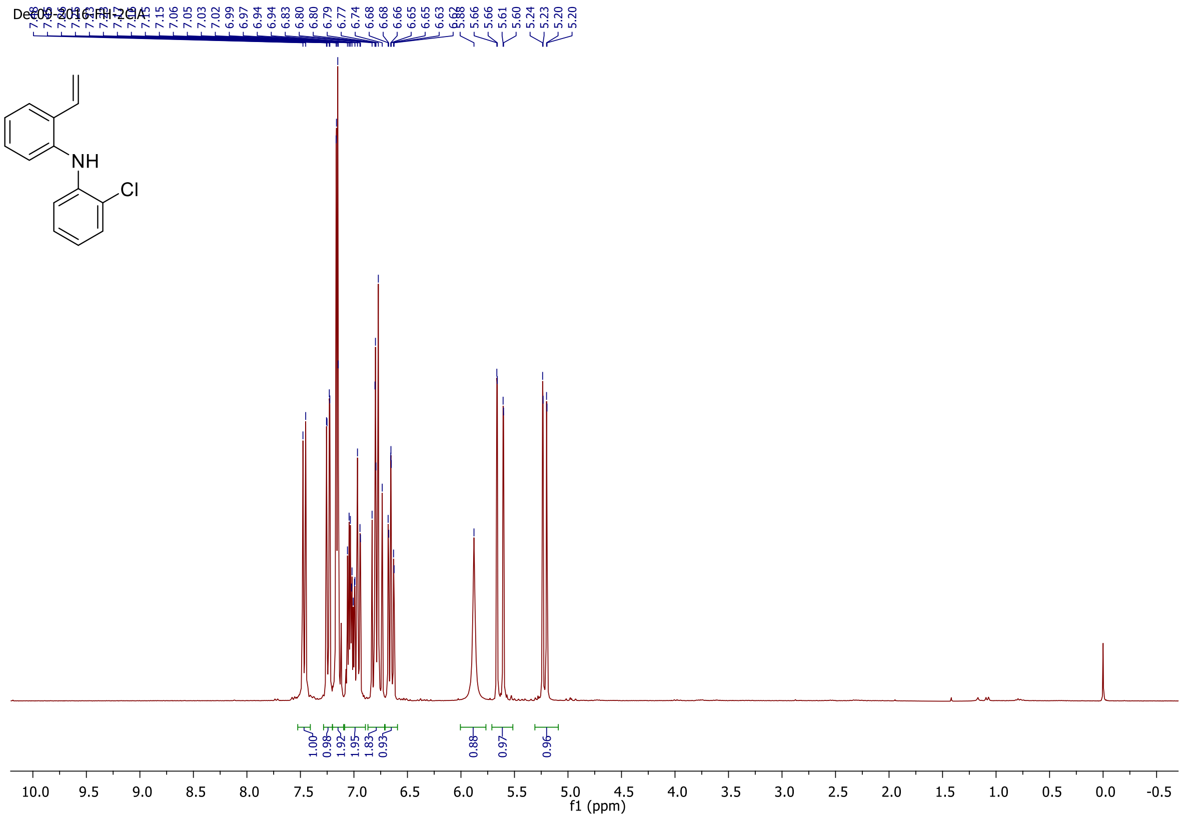

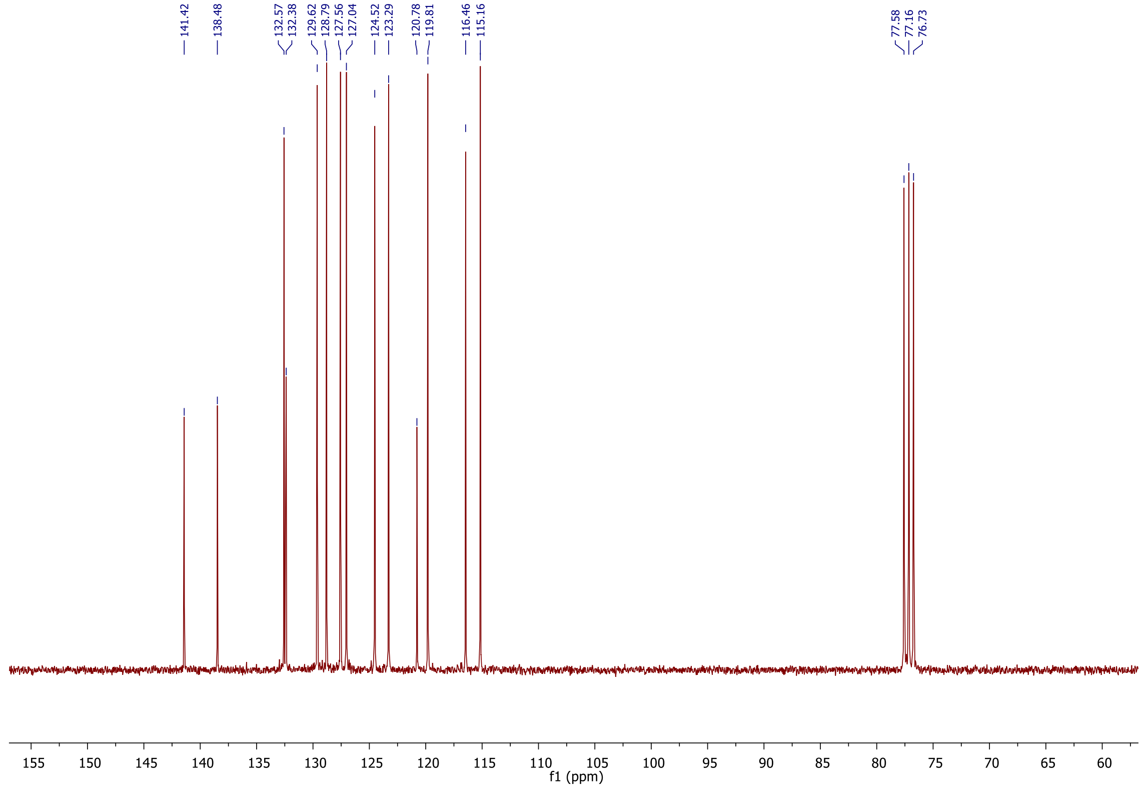

S8 ^{1}H and ^{13}C NMR Spectra of **7g**

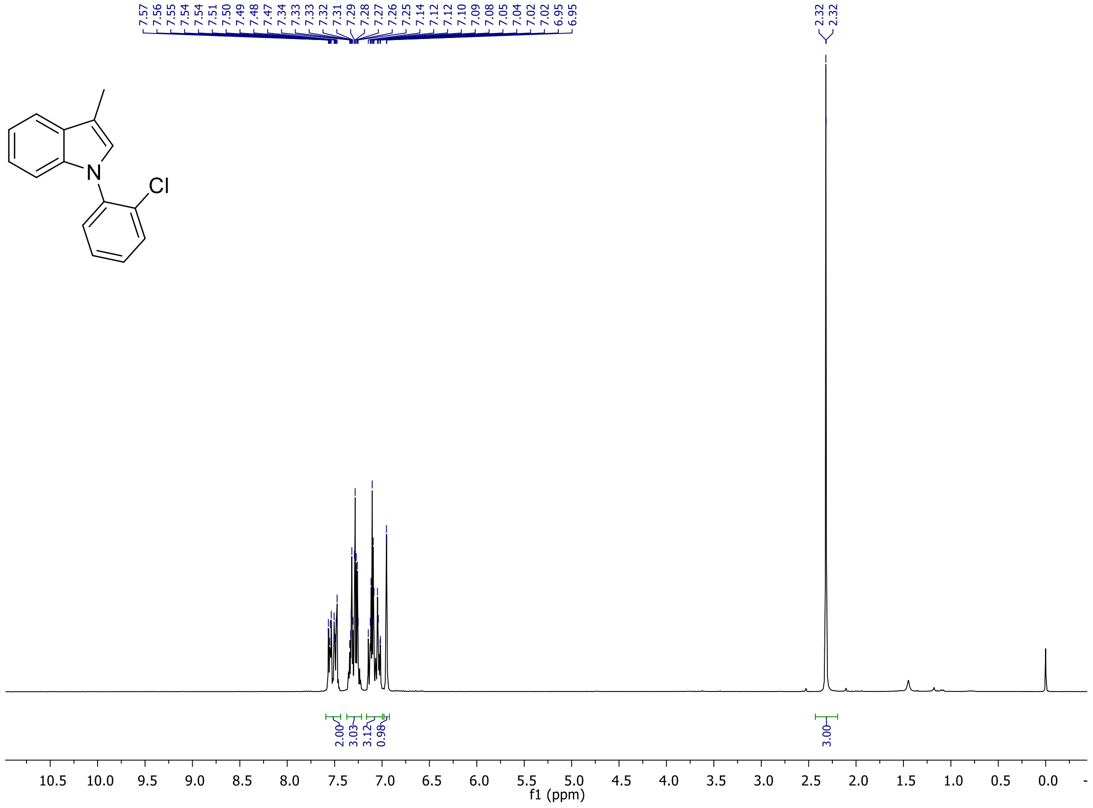

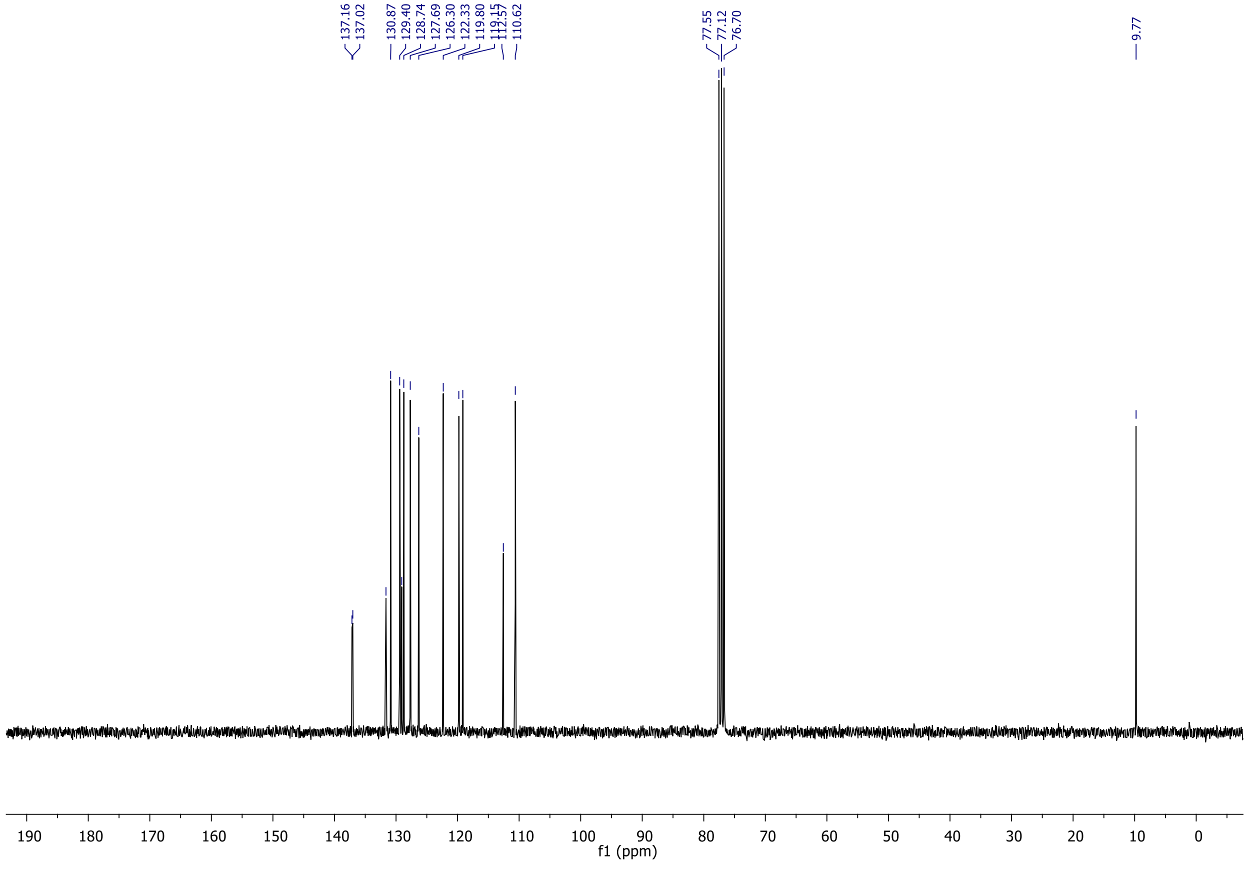

S9 ^{1}H and ^{13}C NMR Spectra of **9a**

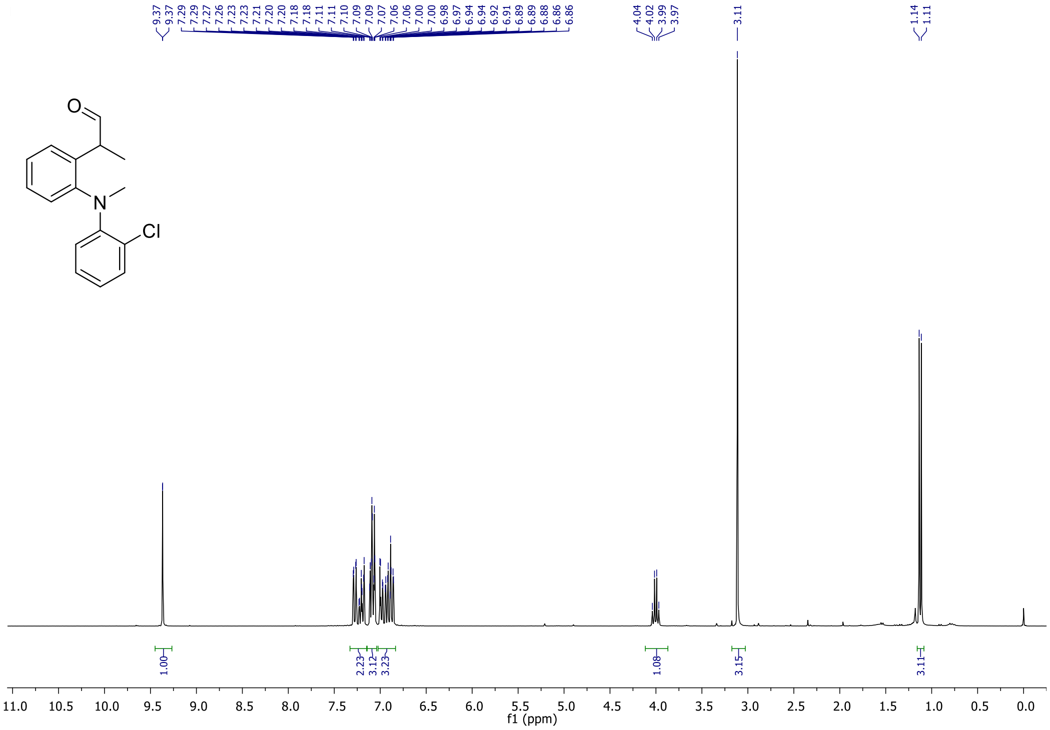

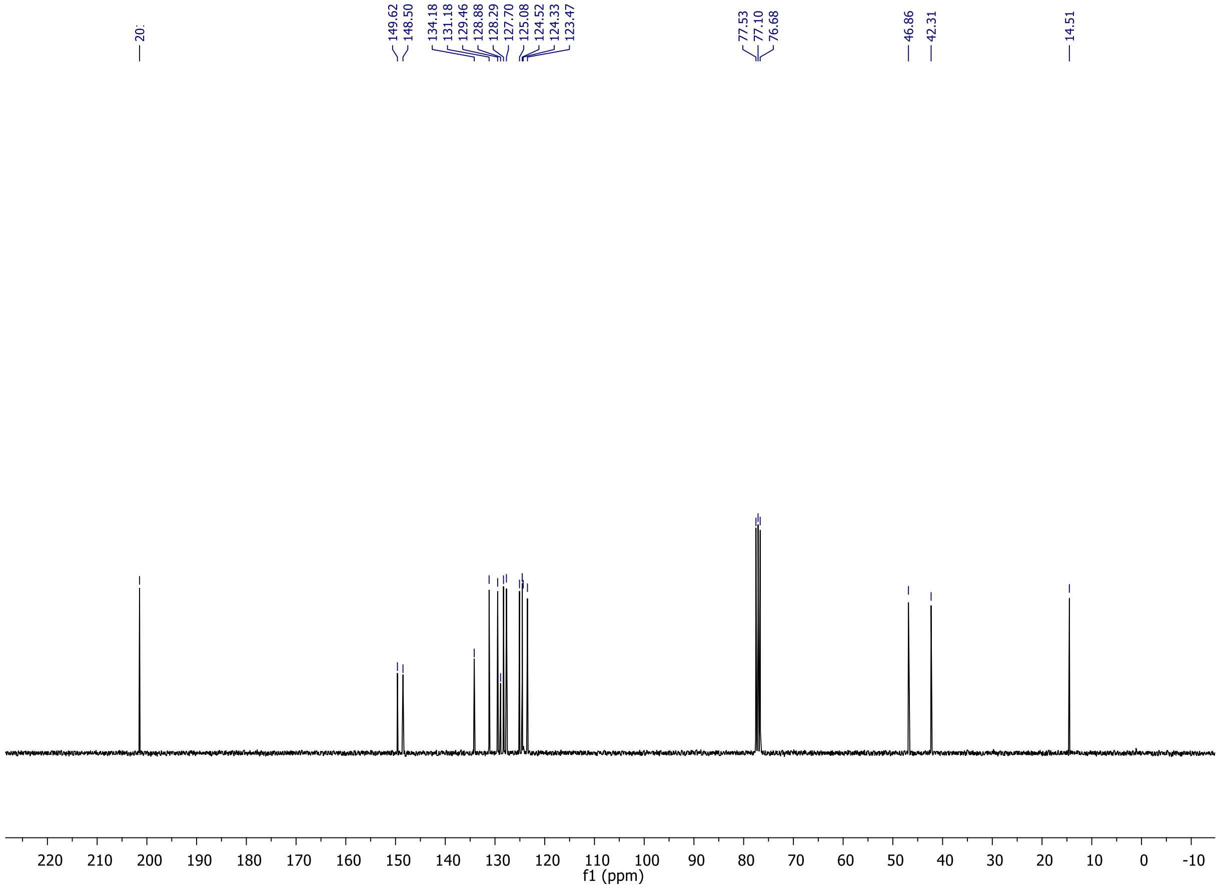

S10 ^{1}H and ^{13}C NMR Spectra of **9b**

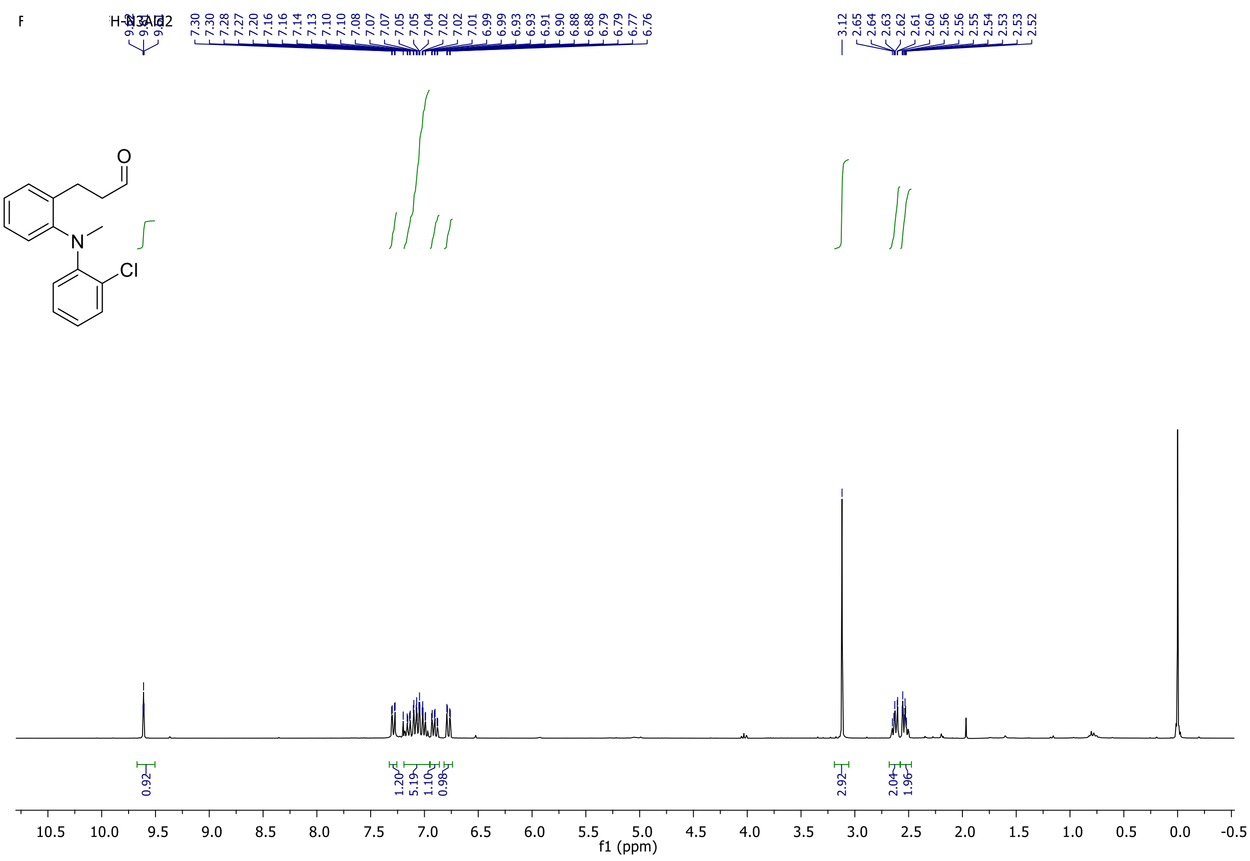

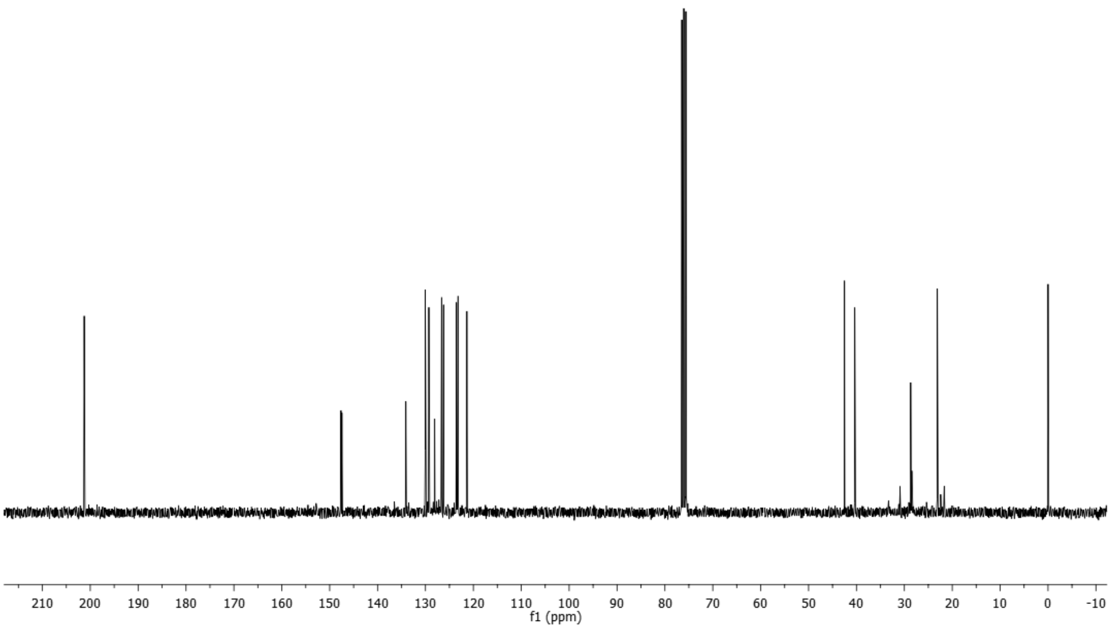

S11 ^{1}H and ^{13}C NMR Spectra **6e**

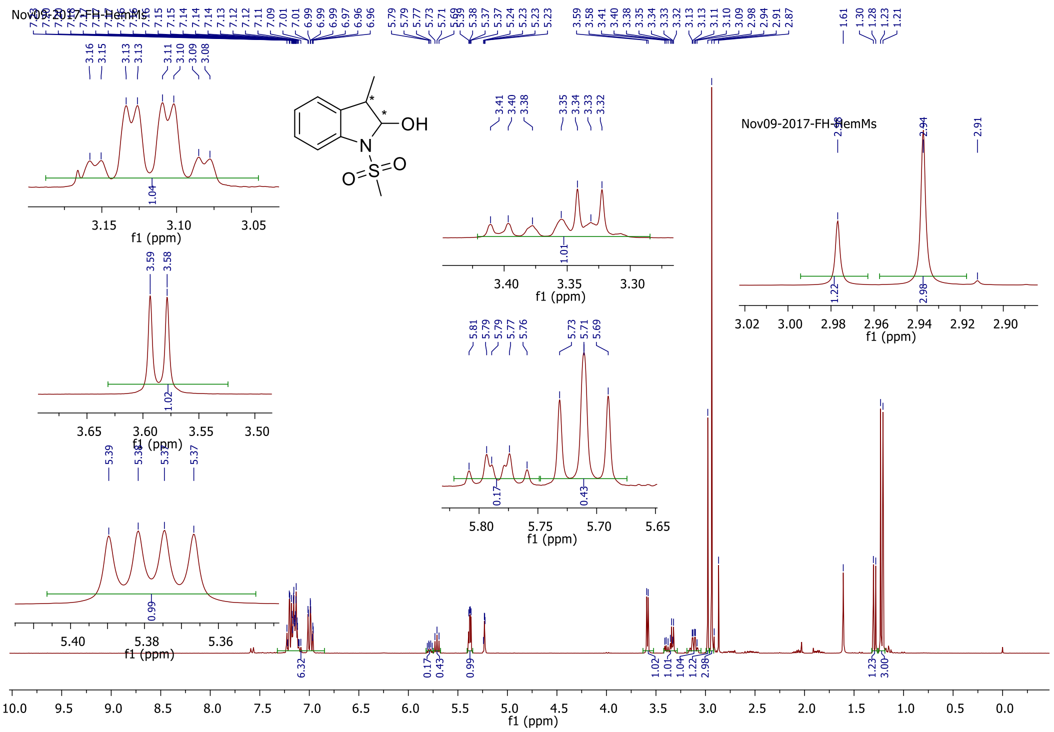

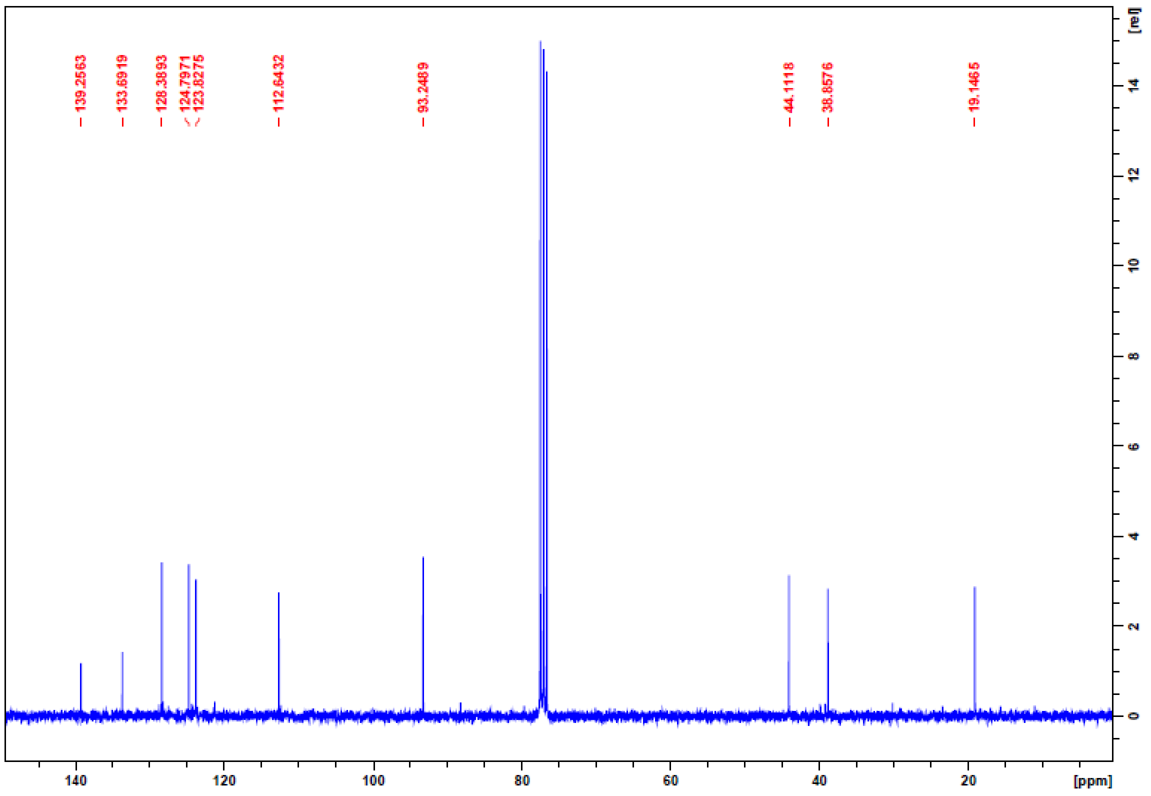

S12 ^{1}H and ^{13}C NMR Spectra of **6f**

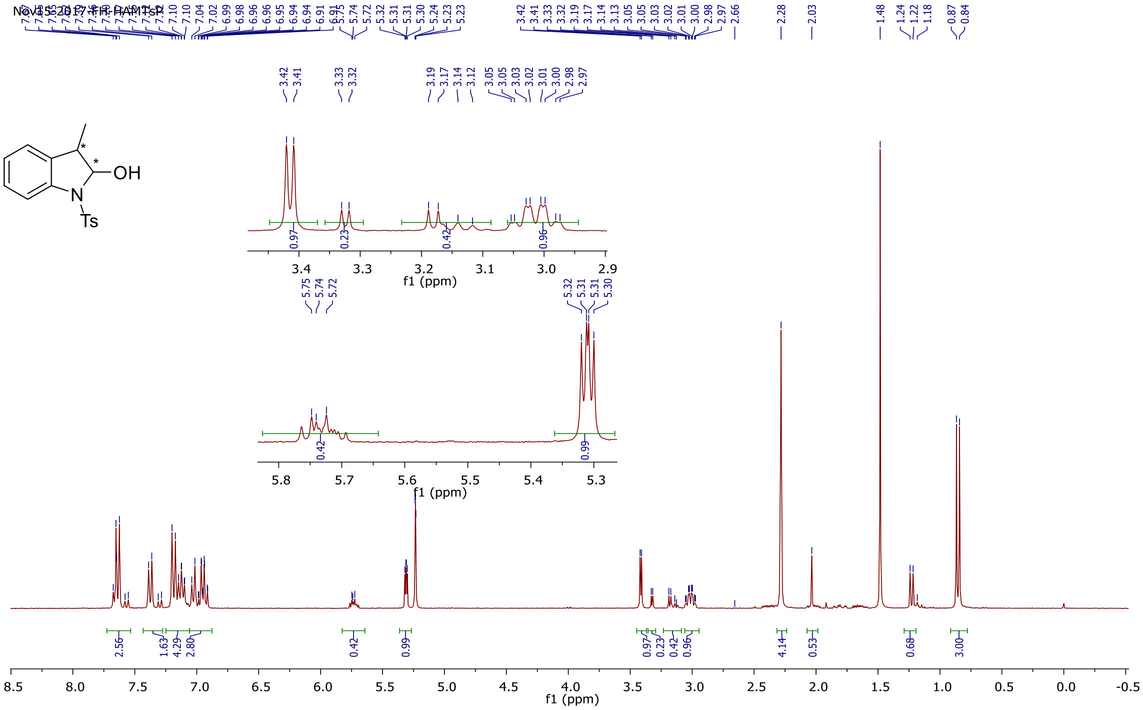

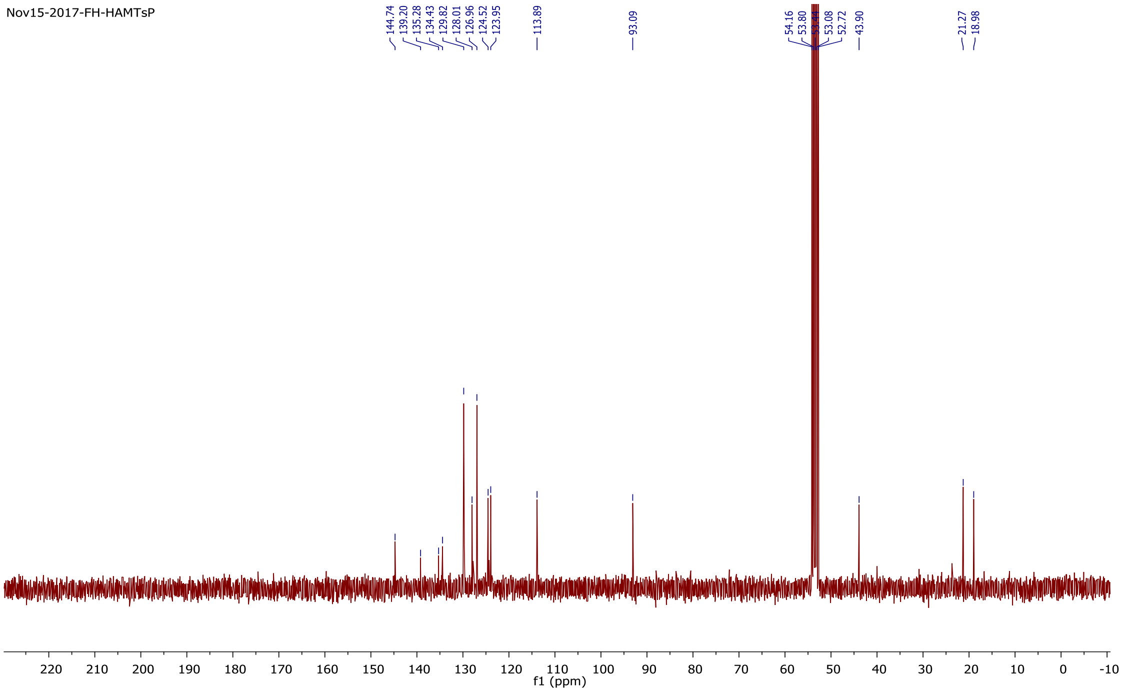

Supplement: Supplementary file 1 [file molecules-27-01074-s001.zip › molecules-1502838-supplementary.pdf]
